# Genome-Wide Identification of Histone Modification Gene Families in the Model Legume *Medicago truncatula* and Their Expression Analysis in Nodules

**DOI:** 10.3390/plants11030322

**Published:** 2022-01-26

**Authors:** Loredana Lopez, Giorgio Perrella, Ornella Calderini, Andrea Porceddu, Francesco Panara

**Affiliations:** 1 Trisaia Research Center, Italian National Agency for New Technologies Energy and Sustainable Economic Development (ENEA), 75026 Rotondella, Italy; loredana.lopez@enea.it (L.L.); giorgio.perrella@enea.it (G.P.); 2 Institute of Biosciences and Bioresources, Consiglio Nazionale delle Ricerche, 06128 Perugia, Italy; 3 Department of Agriculture, University of Sassari, Viale Italia, 39a, 07100 Sassari, Italy; aporceddu@uniss.it

**Keywords:** *Medicago truncatula*, *Medicago sativa*, histone-modifying genes, gene expression, symbiotic nitrogen fixation

## Abstract

Histone methylation and acetylation are key processes in the epigenetic regulation of plant growth, development, and responses to environmental stimuli. The genes encoding for the enzymes that are responsible for these chromatin post-translational modifications, referred to as histone modification genes (HMGs), have been poorly investigated in *Leguminosae* species, despite their importance for establishment and activity of nitrogen-fixing nodules. In silico analysis of *Medicago truncatula* HMGs identified 81 histone methyltransferases, 46 histone demethylases, 64 histone acetyltransferases, and 15 histone deacetylases. MtHMGs were analyzed for their structure and domain composition, and some combinations that were not yet reported in other plant species were identified. Genes have been retrieved from *M. truncatula* A17 and R108 genotypes as well as *M. sativa* CADL and Zhongmu No.1; the gene number and distribution were compared with *Arabidopsis thaliana*. Furthermore, by analyzing the expression data that were obtained at various developmental stages and in different zones of nitrogen-fixing nodules, we identified MtHMG loci that could be involved in nodule development and function. This work sets a reference for HMG genomic organization in legumes which will be useful for functional investigation that is aimed at elucidating HMGs involvement in nodule development and symbiotic nitrogen fixation.

## 1. Introduction

The dynamics and modulation of chromatin changes greatly affects DNA organization and gene expression that, in turn, controls various plant developmental processes and responses to different environmental stimuli [1]. Chromatin is usually organized in nucleosomes, octamer structures that comprise of two copies of histones (H) H2A, H2B, H3, and H4 around which ~146 bp of DNA are compacted [2]. Histone N-terminal tails that protrude from the nucleosomes are mostly enriched in basic-amino acid residues that can be subjected to post-translational modifications (PTMs). The diversity and reoccurrence of these modifications define what is called “the histone code” [3]. The complexity of the histone code relies on the simultaneous presence of single and multiple modifications. Indeed, histone tails can be acetylated, methylated, and/or phosphorylated at various levels (e.g., mono-, di-, and tri-methylation; e.g., the same residue can be both methylated and phosphorylated). Altogether histone PTMs have been linked to different types of downstream processes such as transcription, DNA repair, and chromatin condensation [4].

Histone methylation is one of the major epigenetic mechanisms that can lead to an activation or silencing of gene expression. In Arabidopsis, histone lysine methylation occurs mainly at Lys4 (K4), Lys9 (K9), Lys27 (K27), and Lys36 (K36) of histone H3. These modifications are usually catalyzed by histone methyltransferases (HMTs) [5]. The major class of HMTs are SET domain group (SDG) proteins that, in plants, are classified into four categories, (1) SU(VAR)3–9 groups [including SU(VAR)3–9 homologs (SUVH) and SU(VAR)3–9 related proteins (SUVR)], (2) E(Z) (enhancer of zeste) homologs, (3) TRX (trithorax) groups (TRX homologs and TRX-related proteins), and (4) ASH1 (absent, small, or homeotic discs 1) groups [ASH1 homologs (ASHH) and ASH1-related proteins (ASHR)] [6,7]. Each class of enzymes contains various family members that control the methylation of a specific residue that is based on developmental stages and tissue specificity. The second HMTs subfamily is represented by protein arginine methyl-transferases that contain the Prma domain (PRMTs) [8].

Conversely, histone methylation can be removed by the action of histone demethylases (HDMs), also called erasers. So far, two main HDMs classes have been identified in plants: the KDM1/LSD1-like (HDMAs) and the JmjC domain–containing (JMJs) [9,10]. The two classes work through different mechanisms. For instance, the HDMAs enzymes use amine oxidation for methylation removal, whilst JMJs use hydroxylation [11,12]. Furthermore, the cofactors and the substrates that are associated with these reactions are different: flavin adenine dinucleotide (FAD)-dependent KDM1/LSD1 removes only on di- and mono-methylated but not tri-methylated lysines. JMJs proteins use Fe(II) and α-ketoglutarate (αKG) as cofactors and they can de-methylate lysines at all levels [13].

Histone acetylation and deacetylation occur via the addition and removal of an acetyl CoA group primarily on the lysine residues that are present on H3 and H4 histone tails. The action of presence and absence of acetyl CoA groups is catalyzed by histone acetyltransferases (HATs) and histone deacetylases (HDACs), respectively, which, ultimately, leads to opening and closure of specific chromatin regions that can affect gene expression in response to environmental stimuli [14].

In plants, HATs are organized into four main groups based on domain composition: GNAT and MYST families (HAGs and HAMs, respectively), CBP (HACs), and the TAF_II_250 (HAFs) [15]. HAGs can be further subdivided in three main groups: GCN5, ELP3, and HAT1. Arabidopsis GCN5/HAG1 has been characterized as adaptor of the SAGA complex and modulates several developmental processes, including cell differentiation, leaf and floral organogenesis, and responses to environmental conditions such as light and cold [16]. AtELP3 is part of the elongator protein complex and has a major role in plant immunity and defense [17]. Similarly, AtHAT1 participates in drought responses [18]. The MYST family instead comprises of two members in plants, HAM1 and HAM2, also known as HAG4 and 5 [15]. Phenotypic analysis of Arabidopsis MYST mutants revealed their role in gametophyte development [19].

Plant HDACs have been originally classified in three main classes: The RPD3/HDA1 superfamily, that is based on the homology to the *S. cerevisiae* large RPD3 complex (referred to as HDAs in the following); the Sirtuins (SIR2), hereafter referred to as SRTs; and HD2 (HDTs in the following) which is very specific to plants as no homologs have been identified in animals or fungi [15]. Many members of the RPD3 family have been characterized in plants, revealing key roles in different developmental and physiological mechanisms, including germination, flowering, growth, stress, and temperature responses [20,21,22].

Legumes are an important family of plants providing a main protein source at the global level for both human and animal consumption. In addition, they supply nitrogen to soils thereby reducing the need for chemical fertilization through symbiotic nitrogen fixation (SNF) [23]. There is increasing evidence of the importance of chromatin status and epigenetic regulation in gene function in legumes, particularly concerning nodule-specific genes, which are a characteristic of the species, and they can be silenced by epigenetic marks to obtain tissue specificity and to allow the genes to be active at the proper developmental stages. For instance, Nagymihàly et al. [24] have shown that the expression level of nodule-specific genes in nuclei with different ploidy levels is correlated with the opening of chromatin as well as with reduced H3K27me3 (trimethylation of Lys27 in histone H3) levels in a subset of tested genes. Similarly, Pecrix et al. found that the symbiotic islands showed reduced H3K27me3 levels in nodules compared to roots [25]. Considering that H3K27me3 is a repressive chromatin mark that is deposited by the Polycomb complex, the activation of nodule-specific genes could be due to the opening of the chromatin and the release of H3K37me3-mediated repression during symbiotic cell differentiation [26]. HDACs of the plant-specific HDT type in *M. truncatula* were found to be expressed in the nodule meristem and in the infection zone by a laser capture microdissection (LCM) that was coupled to RNA-seq analysis [27]. A recent functional analysis of the three HDTs by RNAi knock-down that is restricted to nodules demonstrated that they play a key role in primordium formation and nodule development, because both meristem size and cell colonization by bacteria was reduced [28]. Another functional report for the gene *HAC1* in *M. truncatula*, obtained by RNAi downregulation and gene over-expression, showed a range of alterations in plant organ development, supporting a fundamental role for the gene [29]. The expression of the MtJMJC5 gene, a putative Arabidopsis JMJ30/JMJD5 ortholog, was characterized during the circadian rhythm and shown to be oscillating with alternative splicing occurring under cold induction; no functional analysis was provided [30].

A comprehensive analysis of *M. truncatula* genomes identified candidate genes having evolved under positive selection. A total of seven of these genes were histone lysine methyltransferases which suggested that adaptive changes in gene expression may also happen through variation in the methylation enzymes that are involved in epigenetic control [31].

The present paper reports the in silico comprehensive identification of the HMGs families in the *M. truncatula* genome and their expression analysis using data that were obtained from nodules at different developmental stages [32] and in different fractions that were isolated by laser capture microdissection (LCM), [27]. Our results set a reference for HMG genomic organization and form a basis for the functional investigation of HMGs in legumes.

## 2. Results

A total number of 206 HMGs have been identified in the *M. truncatula* A17 genotype and a similar number was identified in the *M. truncatula* R108 genotype (195) and in the *M. sativa* Zhongmu No.1 (194) genotype (see Table 1). This was expected for *M. sativa* Zhongmu No.1 for whom a haploid genome assembly was generated.

A larger number of HMGs (n = 370) was identified in *M. sativa* CADL due to its heterozygous genome with highly divergent haplotypes that were frequently assembled separately [33]. The HMGs of R108, CADL, and Zhongmu No.1 are listed next to the A17 HMGs with the highest similarity score that was obtained by BLAST-P in Table S1. For about 50% of the A17 proteins, it is possible to find two CADL proteins as putative orthologs. However other situations can be observed such as: presence in A17/absence in CADL (17%), and one–one (18%) or one to >2 correspondences (15%) evidencing a possible divergence in the evolution of HMGs. In R108 and Zhongmu No.1, the more frequent ratio is one–one (73% and 58%, respectively). It should be considered that such divergencies might also be due to the differences in the quality of the genome assemblies and annotations.

In A17, the 81 HMTs included 78 SDGs and 3 PRMTs, the 46 HDMs, 34 JMJs and 12 HDMAs; the HATs were 64 subdivided in 51 HAGs, 1 HAM, 11 HACs, and 1 HAF; and the 15 HDACs included 10 HDAs, 2 SRTs, and 3 HDTs. In Table S2 the complete list of *M. truncatula* A17 genes that were identified in our analysis, subdivided by family, is reported.

### 2.1. M. truncatula Histone Methyltransferases (MtHMT)

Histone methyltransferases are classified into two subfamilies: the SDG subfamily that is characterized by the presence of a SET protein domain, and the PRMT subfamily, with PrmA domain as a hallmark.

#### 2.1.1. SDG Subfamily

**Figure 1:**
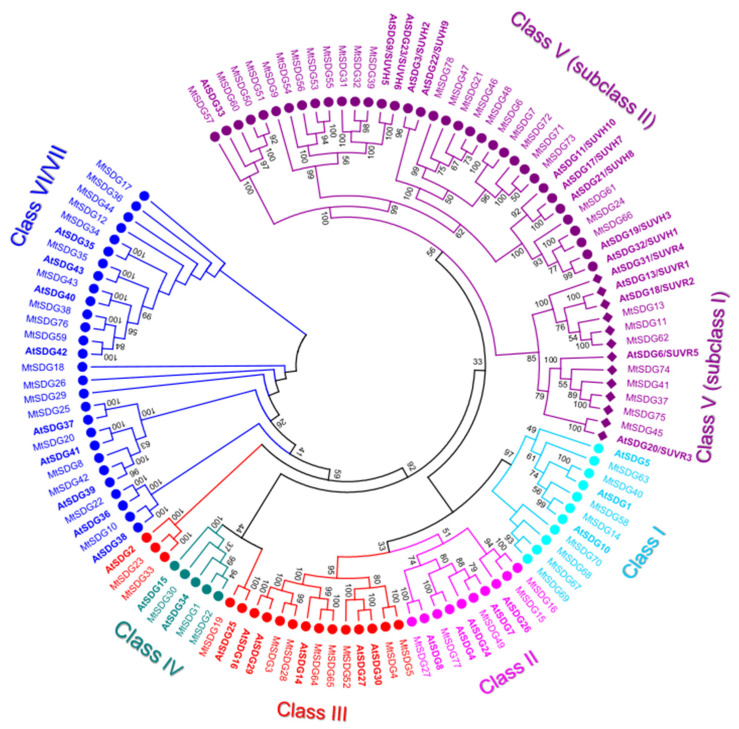
Phylogenetic tree of SDG proteins of *M. truncatula* and Arabidopsis (in bold). The numbers near the tree branches represent bootstrap values.

The 78 *M. truncatula* SDGs are about double in number to that of Arabidopsis (41). All of the 78 MtSDGs were divided into seven classes according to the classification criteria of SDG family in Arabidopsis [34,35] (Figure 1).

**Figure 2:**
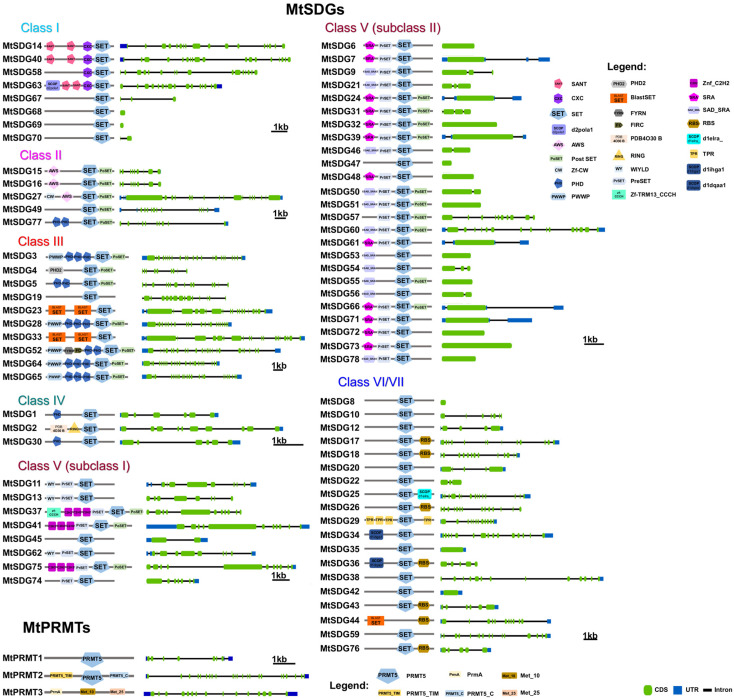
Domain composition and intron-exon structure of *M. truncatula* HMTs.

Domain composition and intro/exon structure of MtHMTs is represented in Figure 2.

In general, the tree topology was coherent with the SDG classes, except there were some minor incongruences for MtHMGs showing homology to AtSDG2 and 25 which are generally included in class III [34,35]. A similar incongruence was observed in *Dendrobium catenatum*, where AtSDG2 was separated from class III SDGs and it was near to AtSDG25 included in Class IV [36]. Indeed, Chen et al. considered their similar substrate specificities and combined the AtSDG2 branch and the neighboring AtSDG25 proteins and categorized them under class M [36].

Class I, E(Z) (enhancer of zeste)-like: *M. truncatula* class I SDG showed the largest number expansion compared to Arabidopsis (2.67 MtSDG to 1 AtSDG). A total of four class I SDGs (MtSDG67–70) derive from tandem duplications in chromosome 8 (Figure S1). However, these could be pseudogenes, as suggested by the short length of the deduced protein (Figure 2). As shown in Figure 2, MtSDG14 and MtSDG40 share a similar intron/exon structure and domain composition with two SANT, one CXC, and one SET domain. Instead MtSDG58 lacks a SANT domain while MtSDG63 has an additional N-terminal domain that is similar to SCOP d2pola1 which is already reported for the beta subunit of *Escherichia coli* DNA Polymerase III holoenzyme. As shown in Figure S1, MtSDG63 and MtSDG14 map in the segmentally duplicated regions in chromosome 1 and 8.

- Class II, ASH1-like: *M. truncatula* Class II SDGs did not show number variation compared to Arabidopsis. All the proteins of the class have SET and post SET domains. MtSDG27, the largest protein of the class, has an additional N-terminal CW-type zinc finger domain as AtSDG8. MtSDG77 has two additional PHD zinc finger domains similarly to AtSDG4. MtSFG15 and MtSDG16, which derive from a tandem duplication on chromosome 1, has an additional AWS domain while MtSDG77 has two additional PHD zinc finger and a structure that is similar to AtSDG4. MtSDG49, instead, lacks the AWS domain.
- Class III, TRX-like: *M. truncatula* Class III SDGs showed a modest number increase when compared to Arabidopsis (Mt:At ratio equal to 1.43:1). MtSDG52 has the same domain composition of AtSDG27 and AtSDG30 including the FYR domain. MtSDG3, MtSDG27, MtSDG64, and MtSDG65 have the same domain composition of AtSDG14, AtSDG16, AtSDG29, and map within segmentally duplicated regions on chromosomes 1, 3, and 7 (Figure S1). MtSDG4 and 5 lack a PWWP domain. MtSDG19 and its orthologous gene AtSDG25 and MtSDG23, MtSDG33, and AtSDG2 identified two separate groups that were both closer to class IV than other class III SDGs. To keep coherence with the original Arabidopsis HMG classification, we included these MtSDG within class III although a classification into the separate class M was also proposed [36].
- Class IV: MtSDG1, 2, and 30 belong to class IV, although tandem duplicated genes MtSDG1 and MtSDG2 show remarkable differences for the intron exon structure and domain composition. Compared to MtSDG1, MtSDG2 has four additional exons at the 5′ and encodes for a larger protein with two additional N-terminal domains. Interestingly, as shown in Table S1, no similar proteins could be found in the other *Medicago* genomes that were analyzed.
- Class V, SU(VAR)3–9-like: *M. truncatula* class V is the largest SDG class with 33 loci. A total of eight are clustered with subclass I Arabidopsis proteins that contain Pre-SET, SET, and Post-SET domains. MtSDG11, 13, and 62 are clustered with AtSDG13, 18, and 31 but all lack the Post-SET domain and have the WIYLD domain at the N-terminal. MtSDG11 and 62 belong to segmentally duplicated regions on chromosomes 1 and 7 (Figure S1). MtSDG37, 41, and 75 have three C2H2 domains similarly to AtSDG6, MtSDG37 also has a zf-CCCH N-terminal domain. MtSDG41 and 75 belong to segmentally duplicated regions on chromosomes 5 and 8 (Figure S1). MtSDG74 is a tandem duplication of the 3′ portion of MtSDG75 harboring only Pre-SET and SET domains. MtSDG45 is more related to AtSDG20. In subclass II, MtSDG24, 61, and 66 are clustered with AtSDG19 and 32 and have the same domain composition except for the PostSET in MtSDG61. There are ten proteins that are clustered with AtSDG3 and 22 and have the same domain composition: SRA-PreSET-SET. Among them are several tandemly duplicated genes: MtSDG71–73 on chromosome 7, MtSDG6 and 7 on chromosome 1, and MtSDG46–48 on chromosome 6. A total of eight proteins are clustered with AtSDG9 and 23 that have the following domain composition: SRA-PreSET-SET-PostSET. MtSDG39 and the tandemly duplicated MtSDG31 and 32 have the expected domain composition. The four tandemly duplicated MtSDG53–56 lack both Pre-SET and Post-SET domains with the exception of MtSDG55 that retained the Post-SET domain. MtSDG9 lacks the Post-SET domain. A total of four proteins are clustered with AtSDG33: MtSDG50, 51, and 60 that have SRA-PreSET-SET-PostSET domains and MtSDG57 lacking SRA.
- Class VI/VII: The 19 MtSDGs that harbor a truncated and/or interrupted SET domain, were classified as belonging to Class VI/VII. Of those, seven have an RBS domain. MtSDG29 has four tetratricopeptide (TPR) domains, MtSDG25 has SCOP d1elra_ domain, and MtSDG34 has SCOP d1ihga1 domain, both are related to TPR and thus are generally involved in protein–protein interactions. MtSDG36 has a SCOP d1dqaa1 domain that is the NAD-binding domain of HMG-CoA reductase in *Homo sapiens*.

#### 2.1.2. Medicago Truncatula PRMT Subfamily

Differently from SDGs, only three PRMTs were found in *M. truncatula* compared to seven of *A. thaliana*. MtPRMT1 and MtPRMT2 have confirmed PF05185 domains by SMART analysis. According to SMART, MtPRMT3 has more likely a PrmA (PF06325) domain that is typical of a ribosomal protein L11 methyltransferase (Figure 2). BLAST-P vs. Arabidopsis proteins assigned a top score (771) to MtPRMT3 alignment with At3g06930 annotated by similarity as a probable histone-arginine methyltransferase named PRMT4B (or PRMT13).

### 2.2. Medicago truncatula HDMs

**Figure 3:**
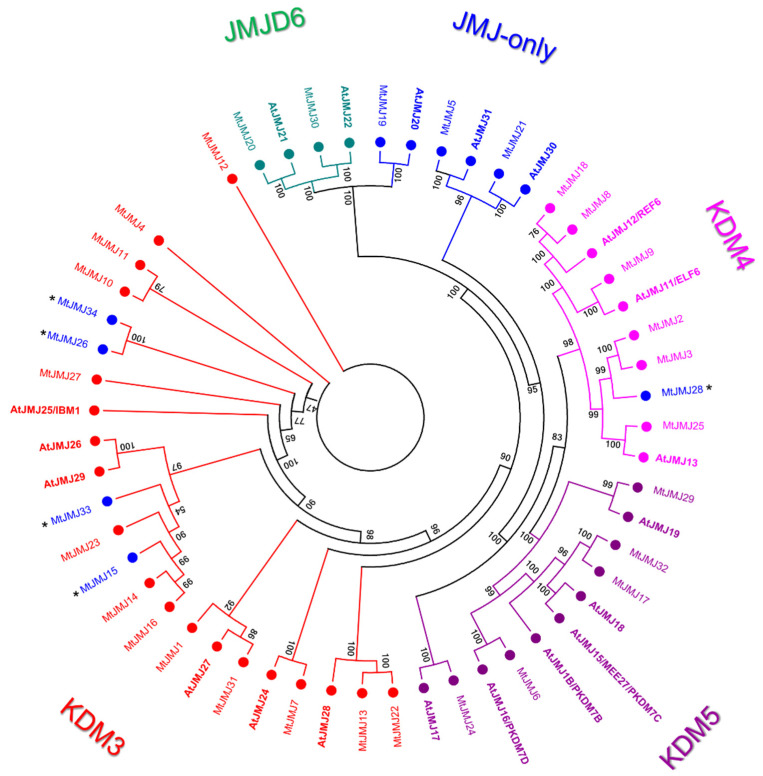
Phylogenetic tree of JMJ proteins of *M. truncatula* and Arabidopsis (in bold). The numbers near the tree branches represent bootstrap values. Asterisks indicate the proteins that underwent domain loss and that could be considered as JMJ-only proteins based on domain composition.

We identified 34 *M. truncatula* proteins that belong to the JMJ family of HDMs compared to 20 that are present in *A. thaliana*. JMJs of Arabidopsis and *M. truncatula* were aligned and a phylogenetic tree was constructed (Figure 3).

As shown in Figure 3, JMJ *M. truncatula* proteins were classified into five different classes according to Arabidopsis JMJ classification: Class I (JMJ-only) with three members in *M. truncatula* corresponding to three in *A. thaliana*, Class II (KDM3) with 17 members in *M. truncatula* and six in *A. thaliana*, Class III (KDM4) with seven members in *M. truncatula* and three in *A. thaliana*, Class IV (KDM5) with five members in *M. truncatula* and six in *A. thaliana*, Class V (JMJD6) with two members in *M. truncatula* and two in *A. thaliana*. MtJMJ15, 26, 33, and 34, classified as KDM3, and MtJMJ28 classified as KDM4; although clustering within the mentioned classes lost the typical domain composition, retaining only the JMJC domain. For this reason, they could be classified as additional members of the JMJ-only class based on domain composition.

**Figure 4:**
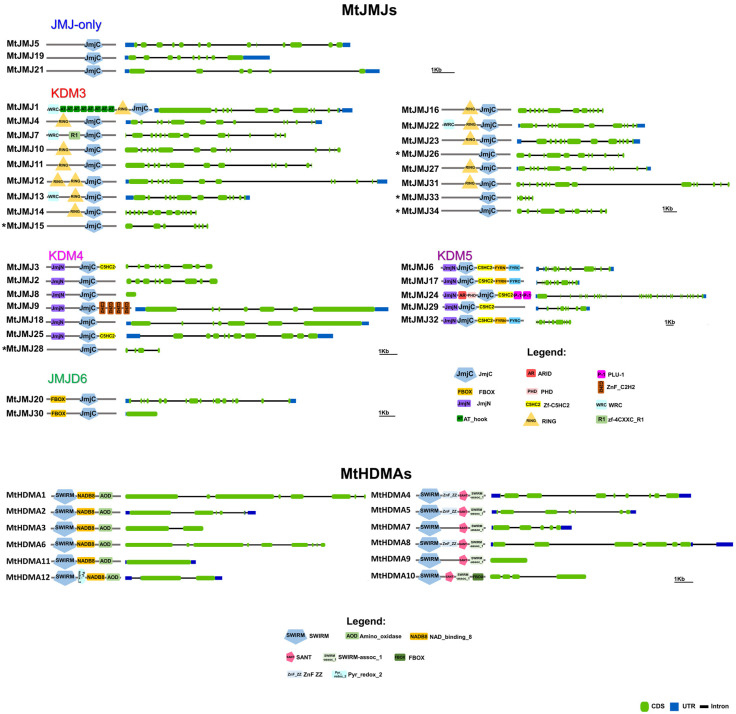
The domain composition and intron-exon structure of *M. truncatula* HDMs. MtJMJs that are indicated with an asterisk belong to the JMJ-only class, based on domain composition, but were put close to their relatives based on phylogenetic analysis to better compare the intron-exon structures.

- JMJ-only: eight proteins possessed only the JMJC domain. Among these, three: MtJMJ5 and MtJMJ21 form a clade with AtJMJ30 and 31 while MtJMJ19 is clustered with AtJMJ20 and is related to JMJD6 Class. The other MtJMJ proteins with only the JMJC domain (15, 26, 28, 33, and 34) as above mentioned do not form a clade with the *A. thaliana* members.
- KDM3: KDM3 class has 13 *M. truncatula* members (1, 4, 7, 10 to 14, 16, 22, 23, 27, and 31), most of them are featured by a JMJC domain at the C-terminal with Ring finger domains (SM000184) ahead of it (Figure 4).

Probably, MtJMJ10 to 12 are tandemly duplicated genes on chromosome 1, and MtJMJ14 to 16 are tandemly duplicated on chromosome 2 (Figure S1). As shown in Figure S1, MtJMJ1 and 31, 13 and 22, 14 and 23 derive from one segmental duplication between chromosomes 1 and 7 and two duplications between chromosomes 2 and 4. Interestingly, MtJMJ1 contains eight tandem repeats of AT_hook domains. MtJMJ1, 7, 13, and 22 have a WRC domain. In addition, only MtJMJ7 presents an R1 domain.

- KDM4: the six MtJMJs of KDM4 class have JMJC and JMJN domains. MtJMJ9 is clustered with AtJMJ11/ELF6 and it is characterized by four tandem repeats of ZnF_C2H2 domain (SM000355). MtJMJ3 and 25 contain a zf-C5HC2 domain (PF02928) at the C-terminal (Figure 4). MtJMJ8 is clustered with MtJMJ18 and AtJMJ12/REF6 but it is a shorter protein of 132 vs. 1543 amino acids that are encoded by a single orf that is probably derived from an aberrant duplication event (Table S2 and Figure 3).
- KDM5: MtJMJ6 is clustered with AtJMJ16/PKDM7D and has the same domain composition (JMJN-JMJC-zf-C5HC2-FYRN-FYRC) of MtJMJ17 and 32. In the same class, MtJMJ24 is clustered with AtJMJ17 and it is the only member with additional ARID, PHD, and PLU-1 domains.
- JMJD6: two proteins that were characterized by a N-terminal F-box (PF00646) domain were found to be related to the JMJD6 class; these proteins, MtJMJ20 and 30 are clustered with AtJMJ21 and 22 respectively (Figure 3 and Figure 4).

### 2.3. Medicago truncatula HDMA Family

A total of 12 genes were identified as members of the HDMA family in *M. truncatula* (Table 1). According to the phylogenetic analysis, six proteins (MtHDMA1, 2, 3, 6, 11, and 12) are clustered with Arabidopsis HDMAs while another six proteins (MtHDMA4, 5, 7, 8, 9, and 10) form a separate group (Figure S2). As shown in Figure S1, MtHDMA1 and 6 belong to segmentally duplicated regions on chromosomes 1 and 3. All MtHDMAs are characterized by the conserved N-terminal SWIRM (PF04433) domain. In addition, MtHDMA1, 2, 3, 6, 11, and 12 are also characterized by the conserved C-terminal Amino_oxidase (PF01593) domain with NAD_BINDING_8 domains ahead of it (Figure 4). In the same group, MtHDMA12 is characterized by the PYR-REDOX_2 domain. MtHDMA4, 5, 7, 8, 9, and 10 harbor the SANT and SWIRM_ASS_1 domains. In the same group, MtHDMA4, 5, and 8, harbor a ZNF_ZZ domain while MtHDMA10 presents a N-terminal FBOX domain (Figure 4).

### 2.4. M. truncatula Histone Acetyltransferase Family (HAT)

In total, 64 HATs were identified in the *M.* *truncatula* genome, a number that is remarkably higher compared to the 12 HATs of *A. thaliana*. The difference is mainly due to HAGs (51 vs. 3) and to a minor extent, to HACs (11 vs. 5).

- HAG: The genomic locations of MtHAGs show that many of them arose probably from tandem duplications: MtHAG4–5, 8–10, 15–17, 24–29, 30–31, 32–33, 34–35, and 39–41. MtHAG4–5 and 24–28 belong to segmentally duplicated regions on chromosomes 1 and 5. A further segmental duplication harbors MtHAG21 and 22 on chromosome 4 and MtHAG30–31 and 32–33 on chromosome 5 (Figure S1). MtHAG44 and MtHAG49 contain the BrD domain and are clustered with AtHAG1 in the GCN5 group (Figures S3 and S4). MtHAG36 is clustered with AtHAG2 in the HAT1 group and has the HAT1 domain. The ELP3 group includes AtHAG3 and MtHAG6 harboring the Elp3 domain. A total of 10 MtHAGs form a cluster with the ELP3 group but lack the Elp3 domain: MtHAG1, 2, 3, 13, 19, 34, 35, 37, 42, and 43. MtHAG34 has an Oleosin domain (PF01277) followed by a transmembrane region and is probably derived from a fusion between an oleosin and a tandem duplication of MtHAG35 (Figures S3 and S4). According to a *M. truncatula* gene expression study [37], MtHAG34 expression is higher in seeds between 16 and 36 dap as expected from Oleosins. A total of seven MtHAGs: 15, 16, 17, 21, 32, 33, and 46 form a cluster that is characterized by Jas-RING-PHD-AT1 domain composition with the exception of MtHAG21 lacking RING and MtHAG17 lacking Jas domains, this cluster is identified as PHD in Figure S3. There are two further clusters that are represented by MtHAG4, 5, 11, 24, 25, 26, 27, 28, 39, 40, 41, and 47 and by MtHAG8, 9, 10, 12, 18, 20, 22, 31, 48, and 50 that shows only the AT1 domain with the exception of MtHAG28 with a KIP1 domain and MtHAG10 with a C-terminal RRM domain. MtHAG7, 14, 23, 29, 30, 38, 45, and 51 all contained the AT1 domain but could not be included in defined clusters probably due to heavier rearrangements in their sequence/structure. Among them, MtHAG30 harbors AA_kinase and SCOP d1gs5a_ domains. Interestingly, the AAK domain was found also in two *Solanum lycopersicum* HAGs: SlNAGS1 and SlNAGS2 that were considered as probable not histone modifiers by the authors [38].
- HAC: AtHACs are characterized by the TAZ-PHD-KAT11-ZZ-TAZ domain composition with the exception of AtHAC2 lacking the N-terminal TAZ domain. Only MtHAC4 and MtHAC8 have all the characteristic domains. All the other MtHACs lack the ZZ domain (Figure S4). A total of five MtHACs (7–11) are tandemly duplicated on chromosome 6 between position 6,665,635 and 6,780,888 and present many rearrangements in sequence and structure. MtHAC2 and 3 are tandemly duplicated on chromosome 3 and have only PHD-KAT11-TAZ domains like MtHAC5, six that are on chromosome 5. MtHAC1 harbors only the KAT11 domain. MtHACs went through several duplication events in the *M. truncatula* genome that generated a series of probably not fully functional histone modifiers (Figures S1 and S4). A phylogenetic tree including MtHACs and AtHACs is displayed in Figure S5.
- HAM and HAF: Only one MtHAM and one MtHAF protein have been identified by our analysis. The domain composition is shown in Figure S4 and is similar to that of AtHAMs and AtHAFs.

### 2.5. M. truncatula Histone Deacetylation Family (HDAC)

MtHDACs are similar in number to those that were identified in other plant species so far. MtHDAs were classified into three classes by phylogenetic analysis against *A. thaliana* HDAs (Figure S6). All MtHDAs display only the HD domain with the exception of MtHDA8 that has a Znf_RBZ domain, a zinc finger domain that is typical of RanBP2 proteins that are responsible for binding to RanGDP (Figure S7). MtHDA3 is probably a truncated tandem duplication of MtHDA4 on chromosome 3 (Figures S1 and S7).

A total of two MtSRTs were identified by our analysis harboring the SIR2 (PF02146) domain (Figure S7). There were also three MtHDTs that were identified by BLASTP of AtHDTs on *M. truncatula* A17 proteome. MtHDT1 and 2 were generated by segmental duplications between chromosomes 2 and 4 (Figures S1 and S8).

### 2.6. Gene Expression Analysis during Nodule Development and in Different Zones of Actively Fixing Nodules

**Figure 5:**
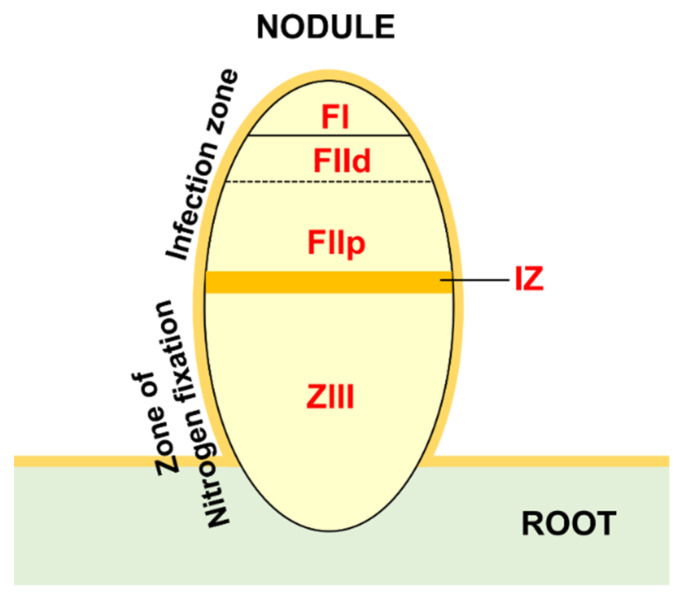
Schematic representation of mature root nodule with indication of the five zones that were sampled by laser capture microdissection (LCM): FI, FIId, FIIp, IZ, and ZIII.

HMGs expression in nitrogen-fixing nodules was evaluated by studying two RNAseq datasets. The ND (nodule development) experiment [32] considered five different stages of nodule development: (1) pre-inoculated roots; (2) root at 4 dpi (days post inoculation), harboring the initial nodule bump during initiation; (3) nodule at 10 dpi (expanding nodule); (4) nodule at 14 dpi (actively-fixing nodule); and 28 dpi (senescing nodule). The LCM (laser-capture microdissection) experiment [27] considered laser-dissected samples from mature nodules: (1) FI, first fraction, the apical region with small cells that were enriched for the nodule meristematic zone; (2) FIId, second fraction distal, the region below FI, distal; (3) the proximal (FIIp) corresponding to ZII (zone II) cells that were undergoing differentiation or infection; (4) the interzone II–III (IZ), which separates ZII from (5) the nitrogen-fixation zone ZIII (see Figure 5 for a schematic representation of different zones).

#### 2.6.1. MtHMTs

In the ND experiment, MtSDG61 showed the highest expression followed by MtSDG14, 23, and 24.

In the LCM experiment, higher expression was observed for MtSDG24 and MtSDG61 followed by MtSDG71, 60, and 36. All MtPRMTs had a high level of expression in at least one sample in both ND and LCM experiments.

**Figure 6:**
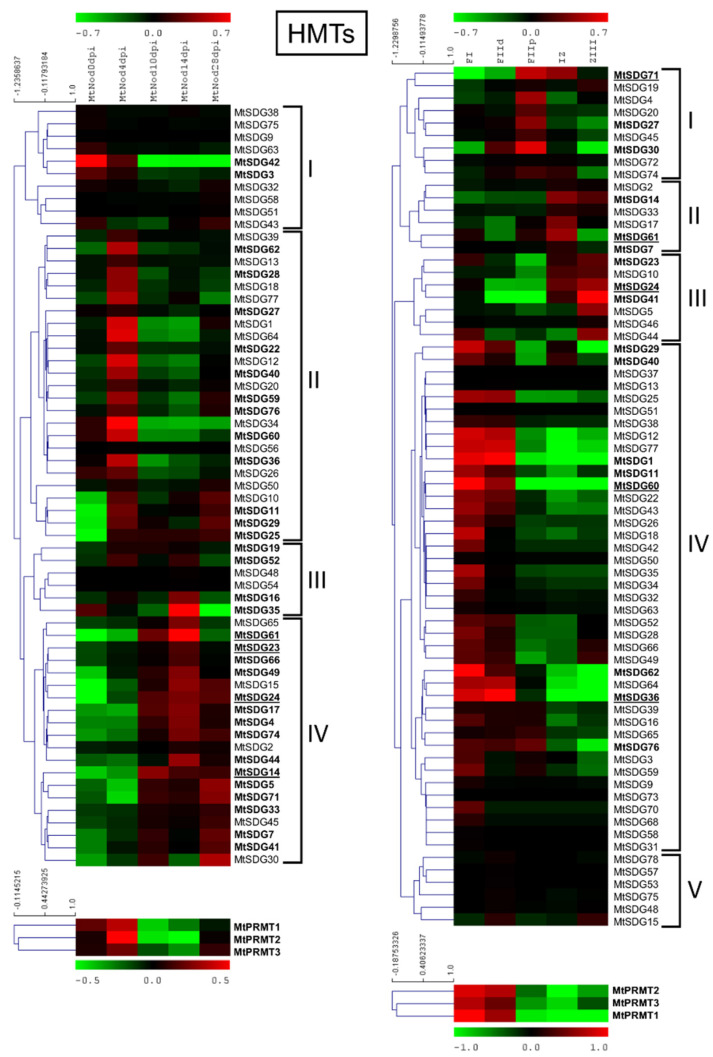
Heat map of *MtHMTs* in the ND experiment (on the left) and the LCM experiment (on the right). The main clusters are indicated by square brackets. In bold are the genes with an average expression above five in the ND experiment and above two in the LCM experiment. Genes with higher expression are underlined.

HMG genes were grouped in clusters based on gene expression patterns among the samples. As shown in Figure 6, four main clusters can be defined for MtSDGs in the ND experiment: cluster I with higher expression in pre-inoculated roots, with a sub-cluster where the expression reactivates in senescing nodules; cluster II with higher expression in the early stages of nodule development; cluster III with higher expression at 4 and 14 dpi; and cluster IV with higher expression in later stages with two sub-clusters with higher levels at 14 dpi and in the senescing nodules.

All MtPRMTs showed a pattern that was characterized by higher expression at four dpi. In the LCM experiment five clusters could be identified: cluster I with higher expression in the proximal fraction II; cluster II in the inter-zone; cluster III in the Zone III where fixation actually occurs; cluster IV with higher expression in FI/distal FII, the meristematic zone of the nodule; and cluster V with a slightly higher expression in FIId. All MtPRMTs showed a pattern with higher expression at FI/distal FII.

#### 2.6.2. MtHDMs

**Figure 7:**
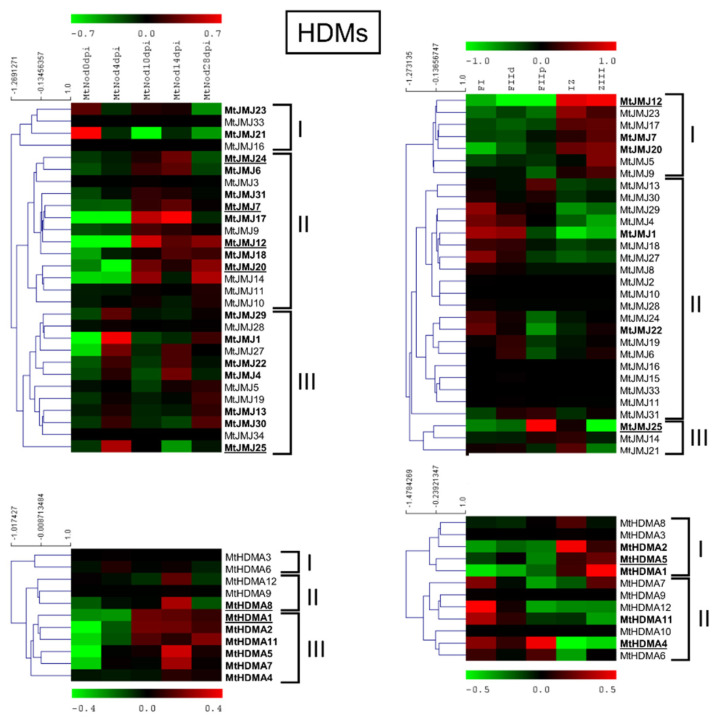
Heat map of *MtHDMs* in the ND experiment (on the left) and the LCM experiment (on the right). The main clusters are indicated by square brackets. In bold are the genes with an average expression above five in the ND experiment and above two in the LCM experiment. Genes with higher expression are underlined.

MtHDM expression analysis in ND and LCM experiment is shown in Figure 7.

MtJMJ12 showed the highest expression level followed by MtJMJ24, 7, 20, and 25 in the ND dataset. MtHDMA1 was the most expressed among the HDMAs, followed by MtHDMA8. MtJMJ25 was the gene with the highest expression followed by MtJMJ12 and for HDMAs: MtHDMA5 and 4 in the LCM dataset.

Cluster analysis of MtJMJs expression variation, as measured in the ND experiment, generated three clusters: cluster I with four MtJMJs that were preferentially expressed in the root before inoculation; cluster II with expression in the nodules 10–14 or 10–28 dpi; cluster III with expression in the young nodules 4 dpi but sometimes with an upturn of expression at 28 dpi (senescent nodules).

Regarding the LCM dataset, the first cluster comprises of genes that were more expressed in IZ to ZIII, in the the second the genes more expressed in FI/FIId, and in the third cluster, FIIp/IZ. Cluster analysis of MtHDMAs identified three clusters: cluster I, two genes that were mainly expressed in root before inoculation; cluster II, three genes that were mainly at 14 dpi; cluster III, six genes with higher expression at 10 to 28 dpi.

#### 2.6.3. MtHATs

**Figure 8:**
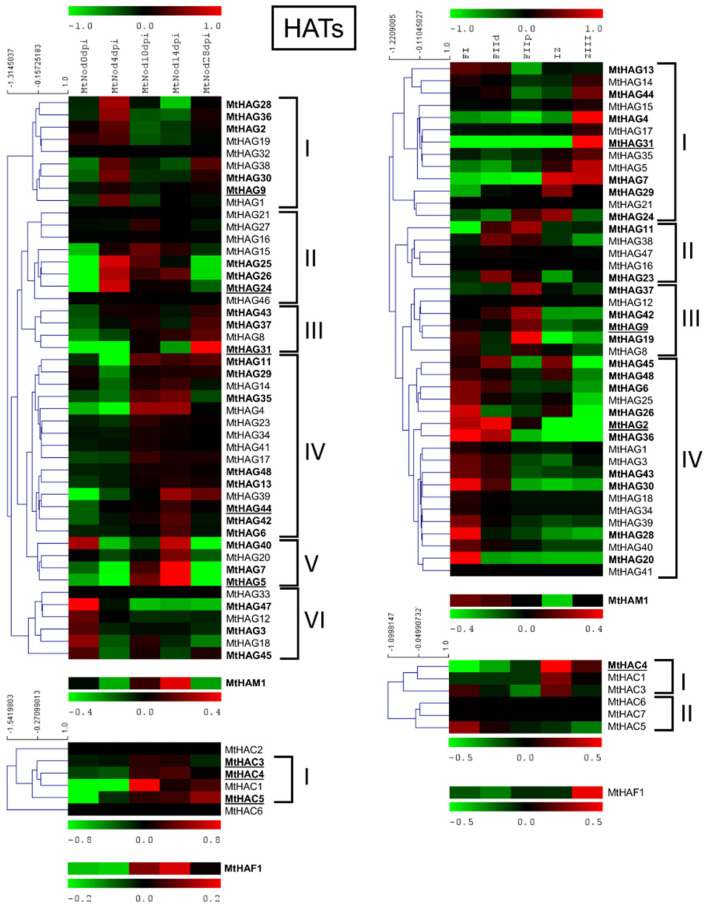
Heat map of *MtHATs* in the ND experiment (on the left) and the LCM experiment (on the right). The main clusters are indicated by square brackets. In bold are the genes with an average expression above five in the ND experiment and above two in the LCM experiment. Genes with higher expression are underlined.

MtHATs expression analysis in the ND and LCM experiment is shown in Figure 8.

MtHAG5 showed the highest expression level followed by MtHAG31, 9, 44, and 24 in the ND experiment. MtHAG31 was the gene with the highest expression followed by MtHAG9 and 2 in the LCM dataset. Among the HACs, MtHAC4 had the highest level of expression in both the ND and LCM, followed by MtHAC3 and 5 only in the ND. MtHAM1 had a high level of expression in both the ND and LCM with higher values at 14 dpi and in FI, FIId. MtHAF1 had a high level of expression in ND and lower in LCM with higher values at 14 dpi and in ZIII.

Cluster analysis of MtHAGs that were based on the expression level in the ND experiment identified five clusters. Cluster I, with the maximum expression at 4 dpi; cluster II, with the maximum between 4 and 14; cluster III identified by genes with the maximum expression at 28 dpi; and finally, cluster IV and V that were identified by genes with the maximum expression at 10–28 and 14 dpi, respectively. Cluster analysis of MtHAGs in LCM generated four clusters with a maximum expression in IZ-ZIII (Cluster I); FIId to FIIp (Cluster II); FIIp (Cluster III); and FI-FIId (Cluster IV). MtHACs showed preferentially higher expression in 10 to 28 dpi samples in ND and formed two clusters in LCM: cluster I with higher expression in IZ, ZIII, and cluster II with higher expression in FI.

#### 2.6.4. MtHDACs

**Figure 9:**
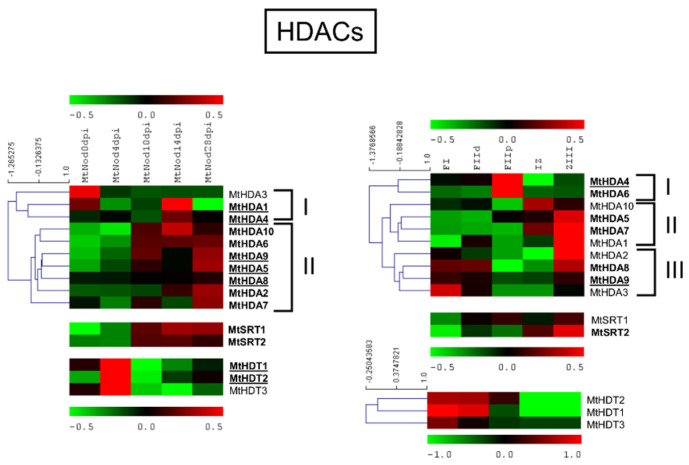
Heat map of *MtHDACs* in the ND experiment (on the left) and the LCM experiment (on the right). The main clusters are indicated by square brackets. In bold are the genes with an average expression above five in the ND experiment and above two in the LCM experiment. Genes with higher expression are underlined.

MtHDACs expression analysis in ND and LCM experiment is shown in Figure 9.

All MtHDACs were expressed in most samples of both the ND and LCM experiment. Among MtHDAs, MtHDA3 showed the lowest level of expression in both experiments with a higher level in the root before inoculation. Most MtHDAs and both MtSRTs were more expressed in 10 to 28 dpi ND samples (Cluster II). All MtHDTs were more expressed at the initial nodule developmental step with high level of expression of MtHDT1 and 2. For MtHDAs, in LCM three clusters were observable with maximum expression in FIIp (cluster I); IZ and ZIII (cluster II); FI and ZIII (cluster III). Both MtSRTs showed slightly higher expression in ZIII. In the LCM experiment, MtHDTs had low levels of expression, mainly in FI-FIId fractions.

## 3. Discussion

### 3.1. Histone Modifier Gene Families in Medicago truncatula

Chromatin-modifiers have been shown to affect various processes in plant development and growth, starting from flowering time, embryo development, as well as response to environmental stimuli [39]. In this manuscript we identified the *M. truncatula* histone-modifying enzymes based on protein domain presence and the degree of homology to Arabidopsis HMGs. In particular, we used A17 and R108 *M. truncatula* genotypes as read outs, together with sequences from genotypes CADL and Zhongmu No.1 of *M. sativa*. Whilst the homologs have been identified based on sequence alignment and synteny analysis with Arabidopsis, currently we do not have direct information about the function of these genes in *M. truncatula*. Therefore, the identification or generation of mutants and over-expressing lines in *M. truncatula* is required to characterize their role.

Altogether we identified 206 MtHMGs in the A17 genotype, while a slightly lower number was scored in R108 (n = 195) as well *M. sativa* Zhongmu No.1 (n = 194). Such genes were distributed in 11 sub-families that were distributed across histone methylation and acetylation groups (Table 1 and Table S1).

For the MtHMTs, in silico analyses revealed a number of SDGs that were almost double of that of Arabidopsis (78 vs. 48) (Figure 1). Interestingly, only the SDG class II showed a number of genes that were similar to Arabidopsis whilst the other classes showed several examples of gene duplication. Among this class we identified homologs of AtSDG4, 8, and 27. AtSDG4 is important to initiate the expression of WUSCHEL (WUS)-RELATED HOMEOBOX 5 (WOX5) by its recruitment to the WOX5 promoter through the chromatin remodeler SEUSS (SEU) [40]. AtSDG8 is mostly involved in H3K36me3 methylation and is linked to the activity of the RNA Pol II in response to *Pseudomonas syringae* [41]. AtSDG27 encodes for ARABIDOPSIS HOMOLOG OF TRITHORAX1 (ATX1) and it is associated with the Polycomb complex to modulate gene expression during seed germination [42].

Within the high number of homologs, class I shows several genes that, despite the presence of the canonical PF00856 domain, encode for much shorter proteins (around 70–100 aa versus 674 aa of a typical MtSDG), suggesting their annotation as putative pseudo-genes (Figure 2).

The majority of MtSDGs show similar protein compositions to those of Arabidopsis and cluster with specific Arabidopsis HMG members (Figure 1). In most cases, several MtSDGs show homology with the same AtSDG making true orthology identification difficult.

Surprisingly, unlike the SDGs, MtPRMTs are lower in number compared to Arabidopsis (3 vs. 7), therefore arguing the relevance and the function of these enzymes in *M. truncatula* (Figure 2). In Arabidopsis, PRMTs function in several developmental processes, primarily flowering and growth [5].

Similar to MtHMTs, MtHDMs are also present in a number that is almost double to those from model species. In particular, MtJMJs were divided into five classes. The JMJ-only clade showed a similar number of members between *Medicago* and Arabidopsis (Figure 3). For example, AtJMJ30 and 31 that show homology to MtJMJ5 and MtJMJ2 are important to demethylate FLOWERING LOCUS C locus at high temperature to prevent early flowering events [43]. AtJMJ20, the homolog of MtJMJ19, is a positive regulator of seed germination that acts redundantly with AtJMJ22 to activate the expression of the gibberellin pathway in a phyB-dependent manner [44]. It will be interesting to assess whether such mechanisms are also conserved in *M. truncatula*. There are five additional proteins with JMJ-only domain structure that are present in *M. truncatula* and derived from genes that clustered within classes KDM3 and KDM4 and that underwent exons loss retaining only the JMJC domain.

MtHDMs Class II (KDM3) includes 13 members of *M. truncatula* compared to the six of *A. thaliana*. Class III (KDM4) shows instead six members in *M. truncatula* and three in *A. thaliana*. Finally, we identified Class IV (KDM5) with five members in *M. truncatula* and six in *A. thaliana,* and Class V (JMJD6) with two members in *M. truncatula* and two in *A. thaliana*. Interestingly, MtJMJ9 is clustered with AtJ/ELF6 while two homologs of the histone methylase REF6 (of which one is a truncated aberrant protein) are identified in *M. truncatula* [45,46] (Figure 3).

The presence of HATs in *M. truncatula* is significantly higher than in Arabidopsis. Our analysis identified 64 MtHATs compared to only 12 in *A. thaliana*. The majority of MtHATs fall in the HAGs class which is almost 20 times higher than in *A. thaliana* (51 vs. 3) (Figure S3). However, a closer look at the data showed several cases of tandem duplication, particularly on chromosomes 1 and 5 (Figure S1). Gene duplication represents one of the types of genomic change that can lead to evolutionary novelties. Indeed, newly discovered functions can arise from the co-option of existing genes, leading the way to the identification of novel functions [47]. Interestingly, at least 10 MtHATs cluster with ELP3 despite missing the canonical ELP3 domain (Figures S3 and S4). Elp3 was first identified as the histone acetyltransferase (HAT) component of the Elp1–Elp6 (Elongator)-complex that associates with RNA polymerase II during transcriptional elongation [48]. A further cluster shows the presence of the AT1 domain. Together with the classic Bromo domain, the AT1 domain is mostly present in the GCN5-like HATs in plants [38] (Figure S4).

Most of the AtHACs are characterized by the presence of the TAZ-PHD-KAT11-ZZ-TAZ domains [49]. Most of the MtHACs lack the ZZ domain, arguing about its functionality (Figure S4). The ZZ domain is relatively small in size and recent work has shown its role in binding the N-terminus of H3, allowing histone acetylation as well as methylation [50]. The HAC1 gene which was reported by [29], corresponding to Medtr7g105680.1 (Mt4.01), although showing a low degree of similarity with AtHAC1 (Blast-P score 121, 41% query cover, 2 × 10^−28^ E-value, 37.5% identity), was not revealed by our analysis because it does not harbor a PF08214 domain.

Unlike the other MtHMG classes, MtHDACs are grouped in a similar number to those that were identified in other plants, including Arabidopsis (Figures S6 and S7). Most of the MtHDACs contain only the HD domain except for MtHDA8 that also presents a zinc finger domain that is typical of RanBP2 proteins that are responsible for binding to RanGDP (Figure S7). RanBP2s are known to play a role in transport between the nucleus and cytoplasm [51]. This indicates that MtHDA8 might diverge from the other MtHDACs. In addition, only two MtSRTs that were identified by our analysis harbored the typical SIR2 (PF02146) domain (Figure S7).

### 3.2. Expression Data Analysis Showed the Possible Involvement of HMGs in Different Aspects of Nodule Development and Function in Medicago truncatula

Nodules are symbiotic organs that are formed by some plant clades, mainly but not exclusively belonging to the *Leguminosae* family [52]. The formation of such structures, dedicated to nitrogen fixation, requires huge transcriptional reprogramming involving the activation and repression of hundreds of genes [26]. HMGs play a role in the control of gene expression by inducing PTMs on histones that are associated with specific DNA regions [4]. *M. truncatula* is a model legume that is broadly studied for its ability to develop indeterminate nodules and perform symbiotic nitrogen fixation. Analysis of the available expression datasets allowed the determination of the behavior of HMGs in nodules during development and in isolated mature nodule fractions [27,32]. Concerning nodule development (ND), HMGs could be subdivided in two main groups: the first one showing higher expression in the initial phases of ND, mainly at four dpi, and a second group that is expressed more later, mainly at 14 dpi when the nodules are actively fixing nitrogen or later. A substantial share of SDGs belonging to ND cluster II, all PRMTs, all HDTs, and a subset of JMJs (ND cluster III) and HAGs (ND cluster I and II) (Figure 6, Figure 7, Figure 8 and Figure 9) belong to the first group and could be involved in the establishment of nodules. The formation and the structure of the nodules resembles that of the roots, in particular the lateral roots (LR) [28]. Histone modifications contribute to the regulation of plant root development [53]. It was demonstrated that histone deacetylase inhibitors block primary root elongation and LR emergence in Arabidopsis, probably influencing the auxin-mediated root development [54]. SKP2B, a negative regulator of LR formation, is also regulated at the epigenetic level through auxin and IAA14/SLR-dependent acetylation of the histone H3 in K9 and K14 associated with the SKP2B promoter [55]. In *M. truncatula*, it was shown that the knockdown of MtHDT expression by RNAi blocked nodule primordium development [28]. Furthermore, in Arabidopsis, several histone methylases and demethylases have been shown to have a role in lateral root formation [56,57]. AtSDG1/CURLY LEAF (CLF), clustering with MtSDG40 (Figure 1), blocks lateral root formation by inhibiting PIN1 expression by depositing H3K27me3 mark on its associated chromatin [58]. AtJMJ12/RELATIVE OF EARLY FLOWERING 6 (REF6), clustering with MtJMJ18 (Figure 3), is also involved in LR formation through modulating the H3K27me3 levels at the chromatin of the PIN1, 3, and 7 genes [59]. AtHDMA3/LDL1/SWP1 negatively regulates LR initiation through direct/indirect transcriptional repression of the LR-promoting factors, such as ARFs and GATA23 [60].

The MtHMGs that are more expressed in mature nodules, (since 10–14 dpi) include SDGs in cluster III and a subset of cluster IV, a subgroup of JMJs in cluster II, most of the HDMAs, HAGs in clusters IV-V and all the other HATs (HAMs, HACs, HAFs), and most of the HDAs and SRTs (Figure 6, Figure 7, Figure 8 and Figure 9). This group could be more specifically involved in the meristematic activity of the indeterminate nodules, in the establishment of symbiotic interaction, and in nodule function and includes primarily MtHMGs with higher expression values. Among them, MtSDG24 and 61 are phylogenetically related to AtSDG32/SUVH1 that methylates ‘Lys-9’ of histone H3 determining the epigenetic transcriptional repression of the associated *loci* [61]. MtHMGs that were more expressed at 28 dpi could be involved in nodule senescence: a subset of cluster IV SDGs, a subgroup of cluster II JMJs, cluster III HAGs, MtHAF1, and a relevant share of HDAs (Figure 6, Figure 7, Figure 8 and Figure 9).

In active nodules, several zones can be identified (LCM experiment, Figure 5). Going from the distal to proximal nodule regions, there is an infection zone where meristematic activity (cell proliferation and differentiation) and infection by nitrogen fixing bacteria occurs and a zone of nitrogen fixation where a mixed population of infected (large) and non-infected (small) cells coexist [27]. Almost all the *HMGs* identified in the LCM experiment showed a preferential expression in specific fractions. A relevant group was expressed in FI-FIId, indicating a possible involvement in meristematic activity and in the maintenance of the meristematic cells niche. This group includes cluster IV SDGs, all PRMTs, cluster II JMJs and HDMAs, cluster IV HAGs, MtHAM, cluster II HACs, cluster III HDAs, and all HDTs (Figure 6, Figure 7, Figure 8 and Figure 9). Interestingly, many genes belonging to this group showed higher expression at four dpi in the ND experiment. Among them, MtSDG60 is related to AtSDG33 that methylates K9 of histone H3 and is involved in the silencing of transposable elements [62].

A second group of HMGs showed higher expression in FIId-FIIp and could have a role in cell differentiation, in the infection process, and in the establishment of the mutual relationships between plant and bacteria. HMGs belonging to this group are cluster I SDGs, cluster III JMJs, cluster II-III HAGs, and cluster I HDAs (Figure 6, Figure 7, Figure 8 and Figure 9).

A third group that is expressed in IZ-ZIII may regulate the nitrogen fixation activity, the maintenance of symbiotic relationship, and the inter-exchanges between infected and uninfected cells. In this group: cluster II-III SDGs, cluster I JMJs, HDMAs, HAGs, and HACs, MtHAF1, cluster II HDAs, and all the SRTs (Figure 6, Figure 7, Figure 8 and Figure 9). Most genes belonging to this group showed higher expression after 10 dpi in the ND experiment, confirming their role in the developed nodules. Among them, MtSDG61 and 24 are phylogenetically related to AtSDG19 and 32. The latter causes a weak reduction of heterochromatic histone H3K9 dimethylation in Arabidopsis [61]. MtSDG23, showing a similar expression pattern, is similar to AtSDG2 involved in H3K4 trimethylation. Loss of function *sdg2* mutants showed smaller plant phenotype due to its possible involvement in the control of transition between the mitotic cycle and cell differentiation [63].

## 4. Materials and Methods

### 4.1. Genomic Sequences

The complete genomic sequence and gene model files of *M. truncatula* (v4.01) [64,65] were downloaded from Phytozome 12 [66]. The genomic sequences of R108 and *M. sativa* CADL (v0.95P) and relative gene models were downloaded from the *M. truncatula* HAPMAP2 site [33]. The *M. sativa* Zhongmu No.1 genomic sequence and relative gene model files were downloaded from [67].

The protein and cds sequences were extracted from the genomic multifasta files using gff2sequence [68].

### 4.2. In Silico Identification and Analysis of HMG Loci

To identify HMGs loci, the Hidden Markov profiles of each gene family were used as a query input for the HMM software against the protein subject datasets of *M. truncatula* (cv. A17 and cv. R108) and *M. sativa* (CADL and Zhongmu No.1). Since for the HDT family no PFAM domain is available, we used Arabidopsis HDTs as query input for blastp searches against the protein datasets. The identified loci were analyzed with the SMART software to characterize the domain composition [69,70]. The cases pointing to two different domains for the same protein region were resolved based on the domain that was associated to the smaller E-value. The intron/exon structures were graphically represented with GSDS 2.0 [71].

### 4.3. Phylogenetic Analysis

The HM protein families from cultivars A17 of *M. truncatula* and Arabidopsis were aligned using ClustalW program in CLC Genomics Workbench version 9.5.2 (Qiagen, Hilden, Germany). A phylogenetic tree was then constructed using a Neighbor-Joining Algorithm and Kimura Protein Substitution Model. Reliability of the internal branching was obtained by a bootstrap test of 1000 replicates.

### 4.4. Synteny Analysis of M. truncatula and Arabidopsis

The analyses of collinearity were performed with the McSCANX software [72] with the default settings. The list of *HMGs* loci, which were collinear to Arabidopsis loci or mapping in segmental duplicated chromosome regions of *M. truncatula,* were extracted from the collinearity file that was produced by McSCANX. The duplicate_classifier was used to classify the remaining duplicates in tandem, proximal, or dispersed. Circos version 0.63 was then used to represent the *HMGs* loci map position and to draw links between collinear loci [73].

### 4.5. Expression Analyses of M. truncatula HMGs in Nodules

*M. truncatula* gene expression experiments were downloaded from the LegumeIP v3 repository [74]. MtHMGs expression patterns were investigated by analyzing two RNAseq experiments: PRJNA213402 which reports a genome-wide expression analysis of laser-captured microsections of developed nodules [27] and PRJNA391316, a developmental time course analysis of root nodules [32].

Normalized gene expression values (FPKM) were calculated by the LegumeIP v3 server that integrates DESeq2 package [75]. Log2 fold changes for each gene were calculated with respect to average FPKM values among the samples. Hierarchical clustering was performed using MeV 4.9.0 [76].

## 5. Conclusions

In-depth in silico analysis of the sequenced genomes and the available expression datasets is instrumental for the comprehension of evolutionary dynamics of target gene families and predicting gene roles in living organisms. Here, we identified genes belonging to the so-called histone-modifying gene families (HMGs) in the reference *M. truncatula* A17 genome in comparison with other *Medicago* genomes (cv. R108 and *M. sativa* “CADL” and “Zhongmu No.1”) and *A. thaliana*. In A17 we identified 81 HMTs, 46 HDMs, 64 HATs, and 15 HDACs. Domain analysis, intron-exon structures, and synteny with Arabidopsis allowed the identification of novel HMGs, the dynamics of gene expansion in the *M. truncatula* genome, and possible neofunctionalizations with introgression of novel domains. Cluster analysis on the gene expression data in the nodule developmental stages and in isolated mature nodule fractions allowed the identification of diversified patterns of expression for HMGs. This led to a comprehensive analysis of the possible roles of the chromatin modifiers in several aspects of SNF. This work sets the basis for future functional evaluations of HMGs in *M. truncatula,* taking advantage of the numerous tools that are available in this model species such as large collections of natural germplasm, artificially-generated mutants, and straightforward protocols for plant transformation and gene editing.

## Figures and Tables

**Table 1:** Gene numbers of each HMG family in *M. truncatula* (A17 and R108) and *M. sativa* (CADL and Zhongmu No.1).

			*M. truncatula*		*M. sativa*	
Type	Family	PFAM	A17 v.4.01	R108	CADL	Zhongmu No.1
HMTs	SDGs	PF00856	78	67	130	68
	PRMTs	PF05185	3	3	6	4
HDMs	JMJs	PF02373	34	29	54	27
	HDMAs	PF04433	12	12	21	12
HATs	HAGs	PF00583	51	53	94	51
	HAMs	PF01853	1	1	2	1
	HACs	PF08214	11	8	22	14
	HAFs	PF09247	1	1	2	1
HDACs	HDAs	PF00850	10	11	19	12
	SRTs	PF02146	2	2	4	2
	HDTs	None	3	8	16	2

## Data Availability

References to all datasets supporting the reported results can be found in the Materials and Methods section.

## Supplementary Materials

The following are available online at https://www.mdpi.com/article/10.3390/plants11030322/s1, Figure S1: Synteny analysis and chromosomal distribution of MtHMGs. Figure S2: Phylogenetic tree of HDMA proteins of *M. truncatula* and Arabidopsis (in bold). Figure S3: Phylogenetic tree of HAG proteins of *M. truncatula* and Arabidopsis (in bold). Figure S4: Domain composition and intron-exon structure of *M. truncatula* HATs. Figure S5: Phylogenetic tree of HAC proteins of *M. truncatula* and Arabidopsis (in bold). Figure S6: Phylogenetic tree of HDA proteins of *M. truncatula* and Arabidopsis (in bold). Figure S7: Domain composition and intron-exon structure of *M. truncatula* HDACs. Figure S8: Phylogenetic tree of HDT proteins of *M. truncatula* and Arabidopsis (in bold). Table S1: List of *M. truncatula* A17, R108 and *M. sativa* CADL, and Zhongmu No.1 HMGs. Table S2: List of *M. truncatula* A17 HMGs.
