# Supplementary material for: Genome-Wide Identification of Histone Modification Gene Families in the Model Legume Medicago truncatula and Their Expression Analysis in Nodules
Source: Plants (Basel). 2022 Jan 26;11(3):322. doi: 10.3390/plants11030322 (PMC8838541; doi:10.3390/plants11030322)
Supplement: Supplementary file 1 [file plants-11-00322-s001.zip › Supplementary_figures_2.pdf]

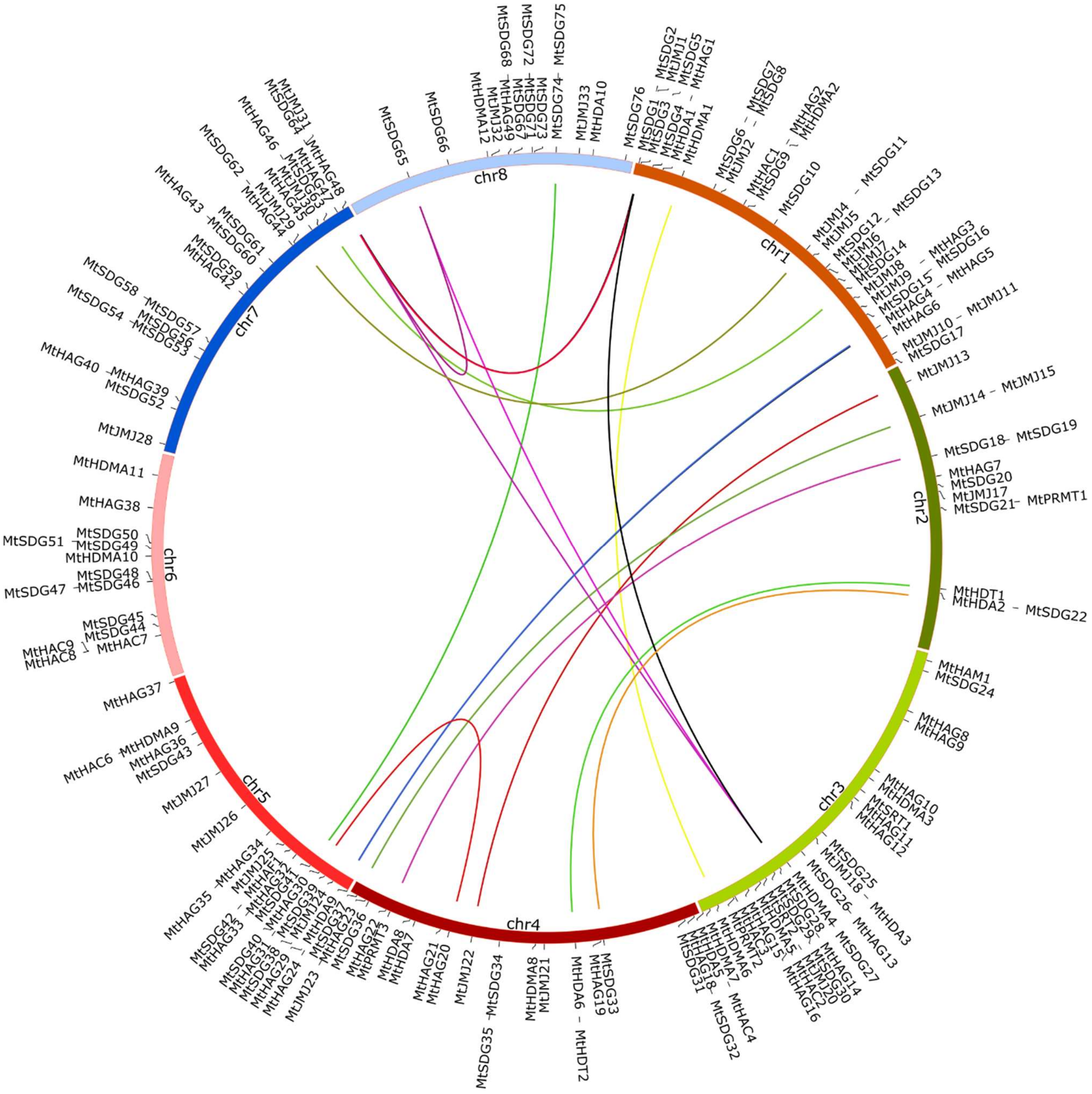

Figure S1. Synteny analysis and chromosomal distribution of MtHMGs.

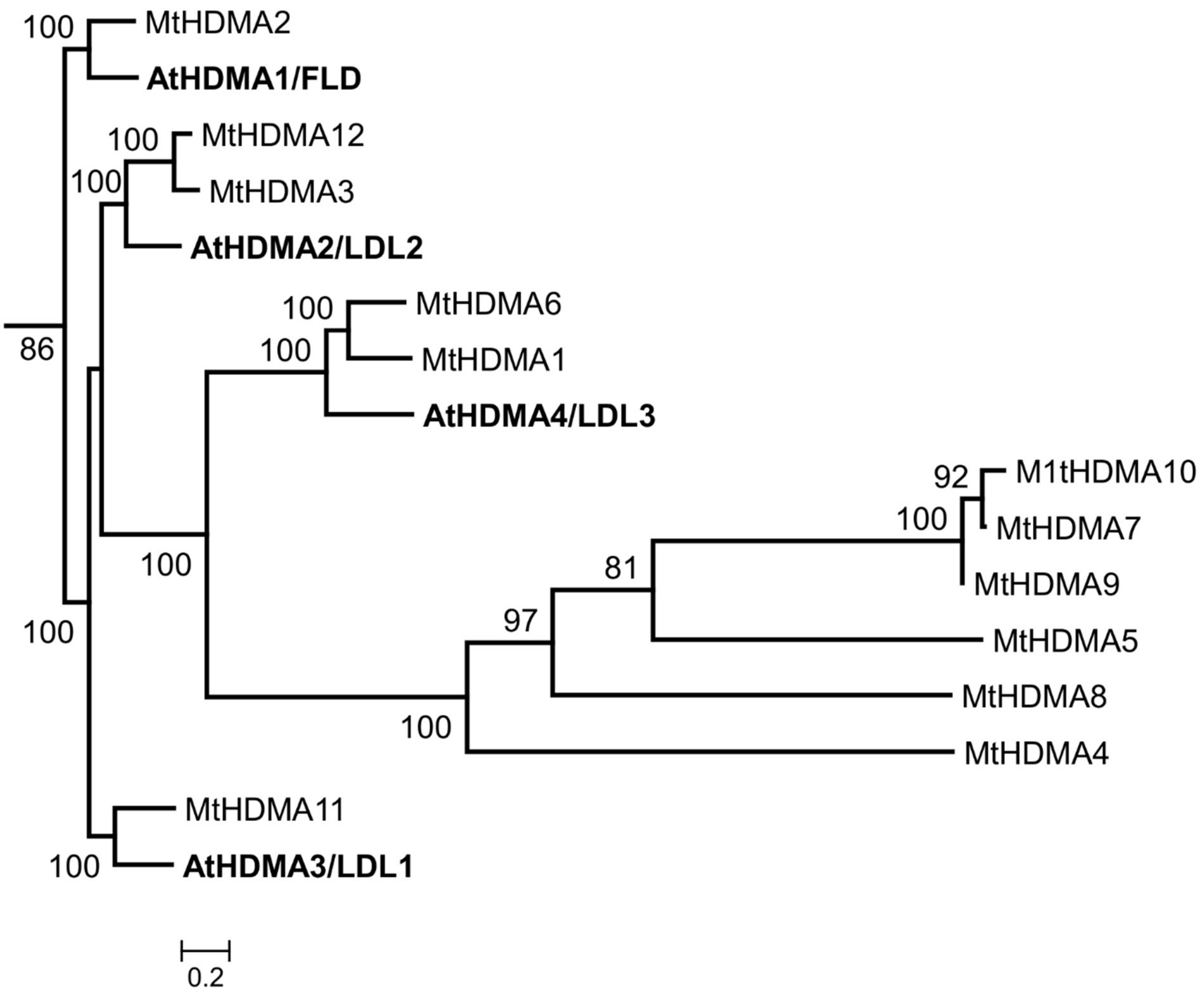

Figure S2. Phylogenetic tree of HDMA proteins of *M.truncatula* and *Arabidopsis* (in bold). Numbers near the tree branches represent bootstrap values.

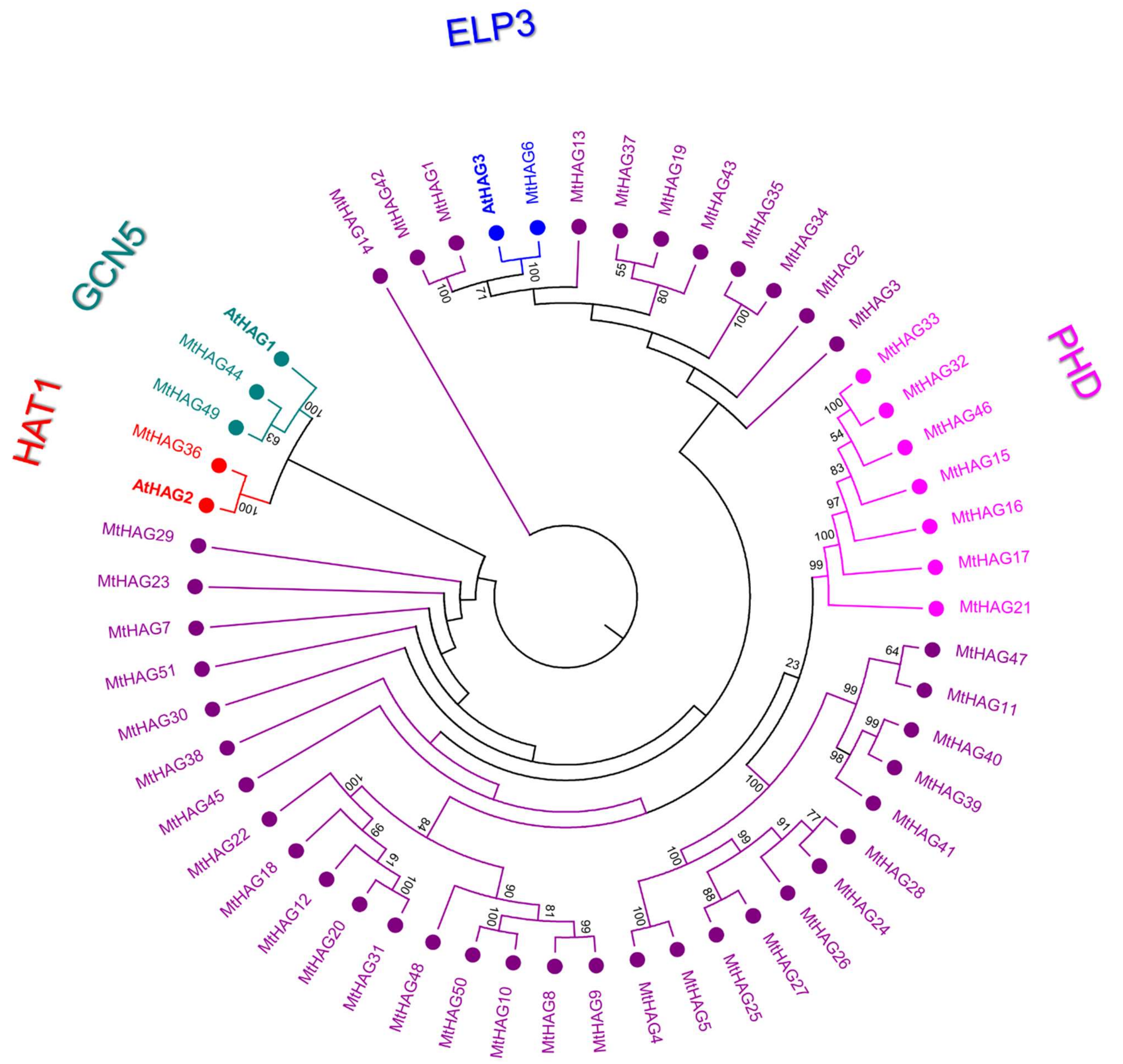

Figure S3. Phylogenetic tree of HAG proteins of *M.truncatula* and *Arabidopsis* (in bold). Numbers near the tree branches represent bootstrap values.

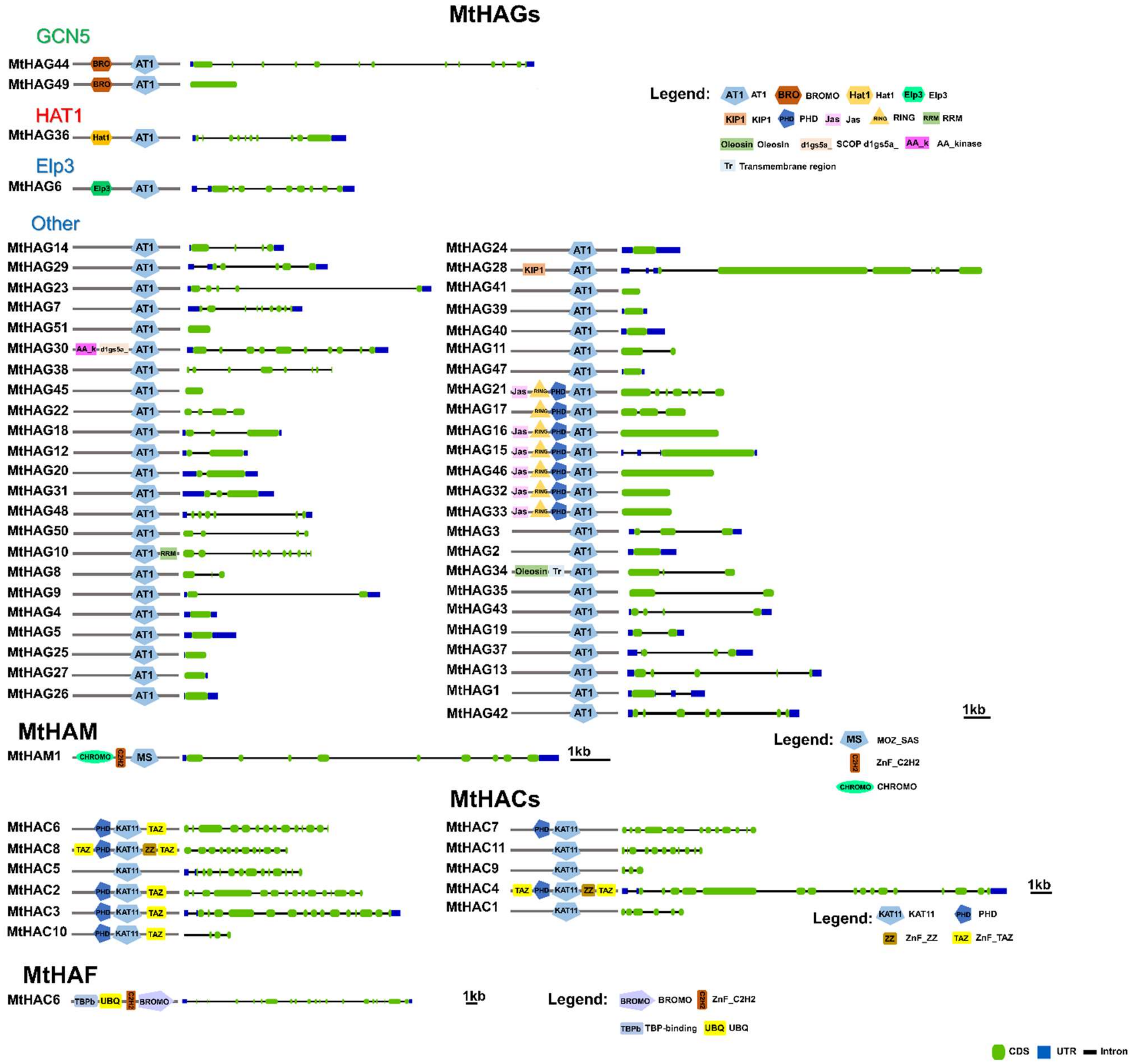

Figure S4. Domain composition and intron-exon structure of *M.truncatula* HATs.

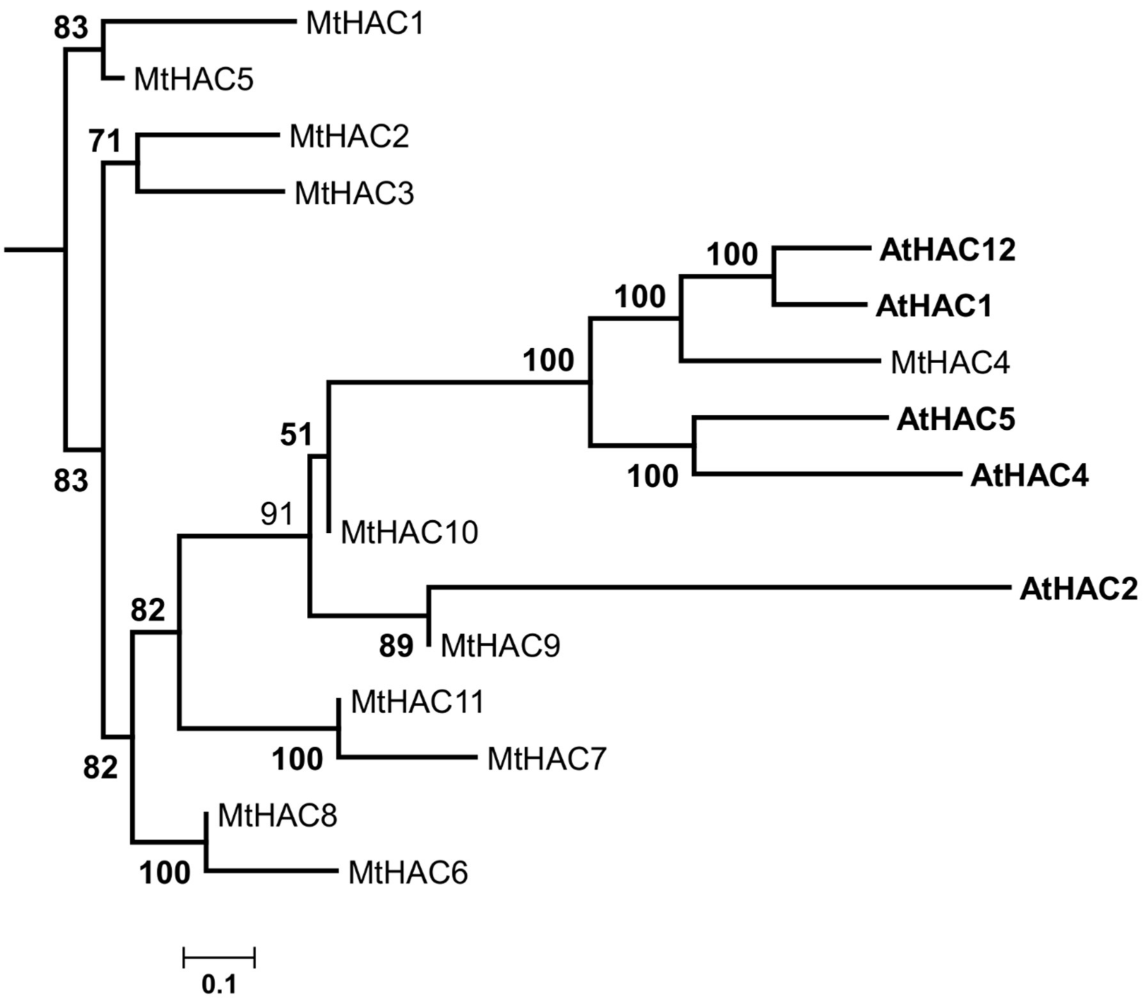

Figure S5. Phylogenetic tree of HAC proteins of *M.truncatula* and *Arabidopsis* (in bold). Numbers near the tree branches represent bootstrap values.

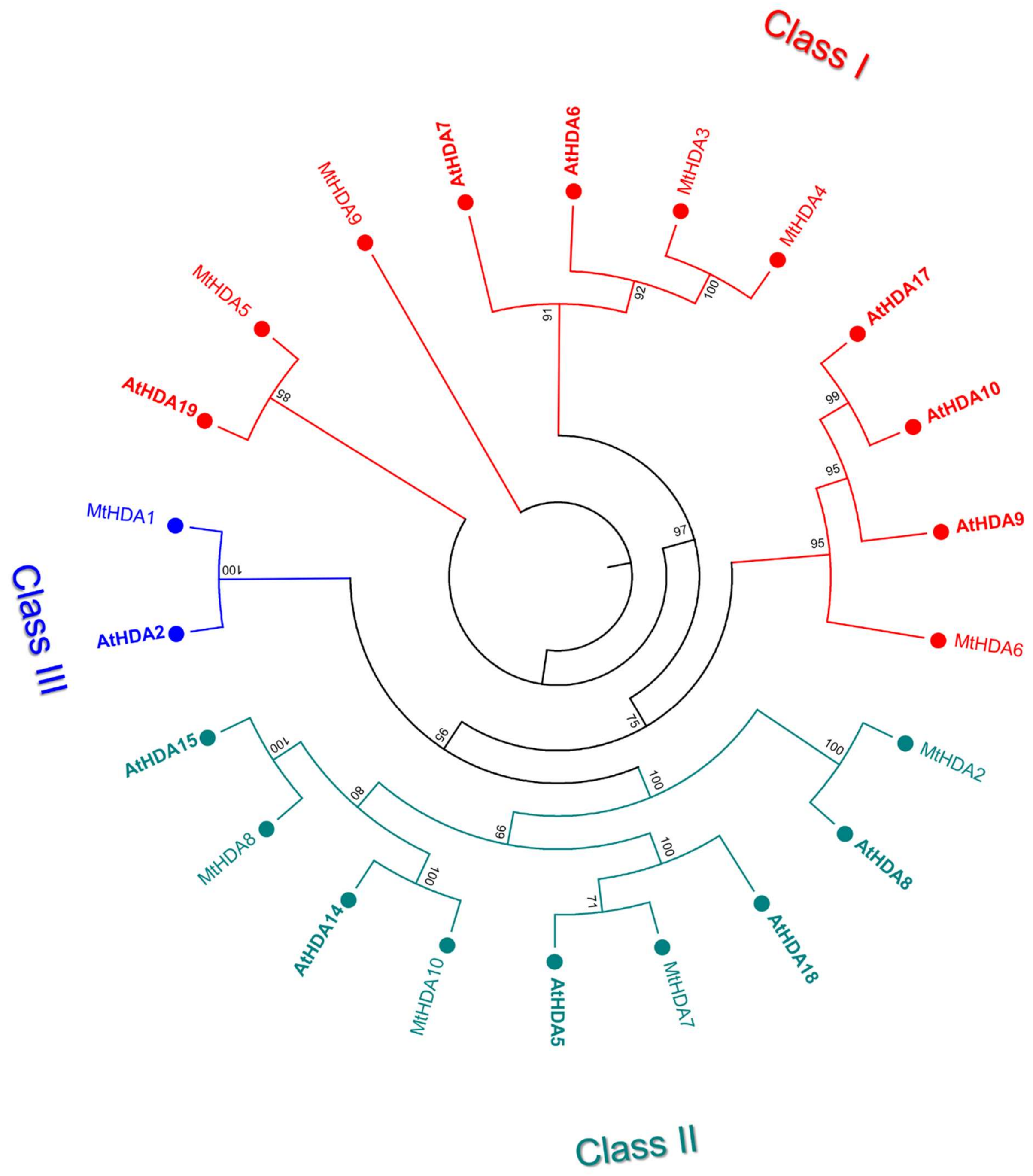

Figure S6. Phylogenetic tree of HDA proteins of *M.truncatula* and *Arabidopsis* (in bold). Numbers near the tree branches represent bootstrap values.

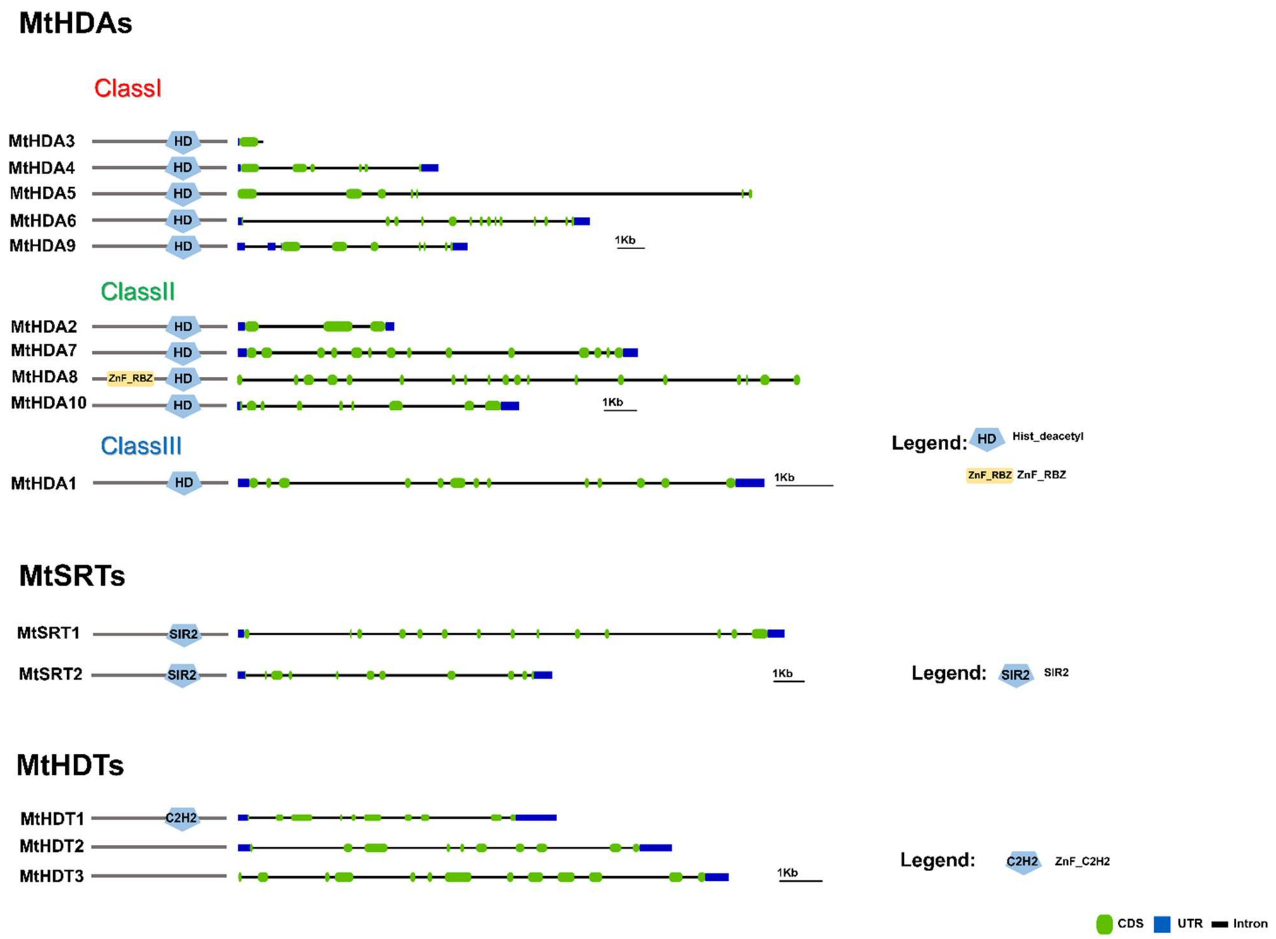

Figure S7. Domain composition and intron-exon structure of *M.truncatula* HDACs.

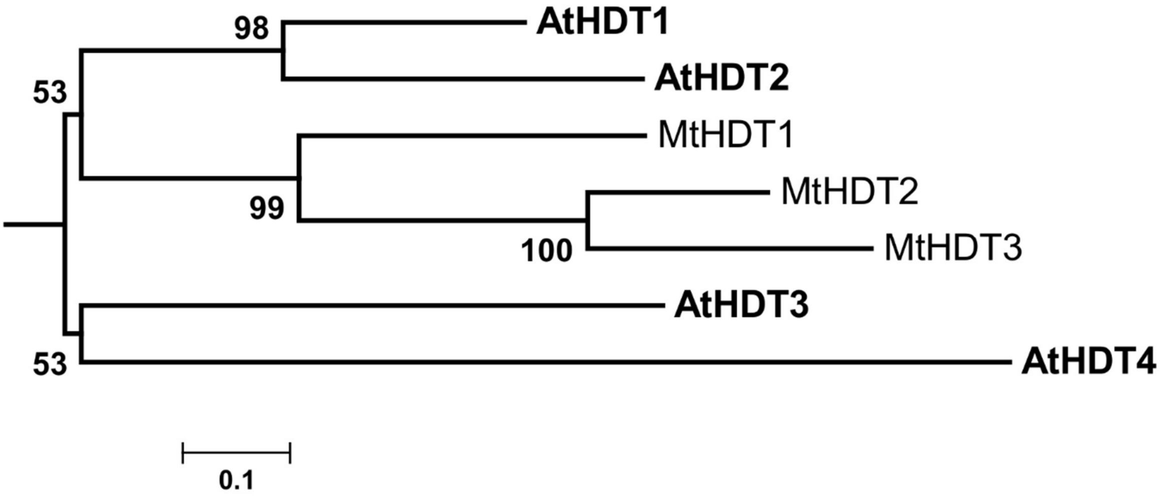

Figure S8. Phylogenetic tree of HDT proteins of *M.truncatula* and *Arabidopsis* (in bold). Numbers near the tree branches represent bootstrap values.
