# Supplementary material for: Genome-Wide Identification of Histone Modification Gene Families in the Model Legume Medicago truncatula and Their Expression Analysis in Nodules
Source: Plants (Basel). 2022 Jan 26;11(3):322. doi: 10.3390/plants11030322 (PMC8838541; doi:10.3390/plants11030322)
Supplement: Supplementary file 1 [file plants-11-00322-s001.zip › Table S2.pdf]

Table S2. List of *M. truncatula* A17 HMGs.

Gene name	*Medicago truncatula* genome v.4.01	Family	Chromosome	Start position	Stop position	CDS (bp)	ORF (aa)
MtSDG1	Medtr1g007670.1	PF00856	chr1	488273	491588	1029	343
MtSDG2	Medtr1g007700.1	PF00856	chr1	498003	503498	1638	546
MtSDG3	Medtr1g008230.1	PF00856	chr1	813490	824184	3105	1035
MtSDG4	Medtr1g012890.1	PF00856	chr1	2706411	2711112	1107	369
MtSDG5	Medtr1g012910.1	PF00856	chr1	2715810	2724787	2016	672
MtSDG6	Medtr1g035400.1	PF00856	chr1	12846365	12848101	1737	579
MtSDG7	Medtr1g035420.2	PF00856	chr1	12857982	12863742	1980	660
MtSDG8	Medtr1g038390.1	PF00856	chr1	14142474	14142866	393	131
MtSDG9	Medtr1g048950.1	PF00856	chr1	18905299	18908044	1692	564
MtSDG10	Medtr1g054665.1	PF00856	chr1	23794876	23799361	909	303
MtSDG11	Medtr1g069570.1	PF00856	chr1	30223098	30229521	2277	759
MtSDG12	Medtr1g077040.1	PF00856	chr1	34376657	34381224	1494	498
MtSDG13	Medtr1g080340.1	PF00856	chr1	35684383	35689448	1806	602
MtSDG14	Medtr1g086980.1	PF00856	chr1	38931314	38941671	2496	832
MtSDG15	Medtr1g098000.1	PF00856	chr1	44071420	44075206	1473	491
MtSDG16	Medtr1g098370.1	PF00856	chr1	44280795	44284611	1482	494
MtSDG17	Medtr1g115980.1	PF00856	chr1	52408798	52417465	1356	452
MtSDG18	Medtr2g035660.1	PF00856	chr2	15050768	15056551	1443	481
MtSDG19	Medtr2g036060.1	PF00856	chr2	15288919	15297613	3639	1213
MtSDG20	Medtr2g045070.1	PF00856	chr2	19607366	19612113	1977	659
MtSDG21	Medtr2g054460.1	PF00856	chr2	23252609	23254150	1404	468
MtSDG22	Medtr2g088820.1	PF00856	chr2	37447568	37454133	1467	489
MtSDG23	Medtr2g089080.1	PF00856	chr2	37577237	37590737	7491	2497
MtSDG24	Medtr3g011440.1	PF00856	chr3	2824195	2828442	1995	665
MtSDG25	Medtr3g073000.1	PF00856	chr3	32855213	32861120	1428	476
MtSDG26	Medtr3g082690.1	PF00856	chr3	37307346	37311457	1494	498
MtSDG27	Medtr3g088625.1	PF00856	chr3	40333388	40348349	6552	2184
MtSDG28	Medtr3g091310.1	PF00856	chr3	41524903	41534178	3192	1064
MtSDG29	Medtr3g093440.2	PF00856	chr3	42700289	42708495	2337	779
MtSDG30	Medtr3g095840.1	PF00856	chr3	43798849	43802903	1173	391
MtSDG31	Medtr4g010550.1	PF00856	chr4	2496336	2497880	1395	465
MtSDG32	Medtr4g010830.1	PF00856	chr4	2511109	2514051	2943	981
MtSDG33	Medtr4g045743.1	PF00856	chr4	15584649	15601538	7977	2659
MtSDG34	Medtr4g082140.1	PF00856	chr4	31885978	31887837	1623	541
MtSDG35	Medtr4g082305.1	PF00856	chr4	32029066	32031775	1473	491
MtSDG36	Medtr4g128240.1	PF00856	chr4	53373038	53384921	1920	640
MtSDG37	Medtr4g132610.1	PF00856	chr4	55443635	55449188	3144	1048
MtSDG38	Medtr5g010630.1	PF00856	chr5	2885090	2885915	315	105
MtSDG39	Medtr5g013420.1	PF00856	chr5	4256679	4261177	3276	1092
MtSDG40	Medtr5g016870.1	PF00856	chr5	6095959	6106673	2592	864
MtSDG41	Medtr5g018850.3	PF00856	chr5	7042376	7051901	4524	1508
MtSDG42	Medtr5g027020.1	PF00856	chr5	11193468	11195062	1074	358
MtSDG43	Medtr5g076640.1	PF00856	chr5	32701993	32706219	1467	489
MtSDG44	Medtr6g023320.1	PF00856	chr6	8121326	8129384	1506	502

MtSDG45	Medtr6g027690.1	PF00856	chr6	9665535	9669113	1275	425
MtSDG46	Medtr6g043960.1	PF00856	chr6	15167317	15168836	1458	486
MtSDG47	Medtr6g044000.1	PF00856	chr6	15181400	15181918	519	173
MtSDG48	Medtr6g044690.1	PF00856	chr6	15474733	15476436	1704	568
MtSDG49	Medtr6g059310.2	PF00856	chr6	20350572	20359719	1050	350
MtSDG50	Medtr6g061200.1	PF00856	chr6	21239335	21241419	1998	666
MtSDG51	Medtr6g061270.1	PF00856	chr6	21287289	21289373	2085	695
MtSDG52	Medtr7g021365.1	PF00856	chr7	6749832	6764175	3210	1070
MtSDG53	Medtr7g445530.1	PF00856	chr7	15429010	15430551	1542	514
MtSDG54	Medtr7g445600.1	PF00856	chr7	15448550	15450069	1071	357
MtSDG55	Medtr7g445710.1	PF00856	chr7	15487405	15489036	1632	544
MtSDG56	Medtr7g450730.1	PF00856	chr7	16942138	16943730	1518	506
MtSDG57	Medtr7g052480.1	PF00856	chr7	18442593	18447560	1671	557
MtSDG58	Medtr7g055660.1	PF00856	chr7	19084301	19092927	2199	733
MtSDG59	Medtr7g076590.1	PF00856	chr7	28843776	28851809	1644	548
MtSDG60	Medtr7g084090.1	PF00856	chr7	32418096	32426805	2274	758
MtSDG61	Medtr7g088370.1	PF00856	chr7	34415516	34420153	2118	706
MtSDG62	Medtr7g098390.1	PF00856	chr7	39370159	39376536	2142	714
MtSDG63	Medtr7g109560.1	PF00856	chr7	44824669	44831064	2295	765
MtSDG64	Medtr7g117355.1	PF00856	chr7	48479850	48487877	2895	965
MtSDG65	Medtr8g027725.2	PF00856	chr8	10031702	10042018	2850	950
MtSDG66	Medtr8g042750.1	PF00856	chr8	16510603	16517093	2067	689
MtSDG67	Medtr8g064240.1	PF00856	chr8	26939638	26943145	357	119
MtSDG68	Medtr8g064250.1	PF00856	chr8	26941720	26942040	321	107
MtSDG69	Medtr8g064310.1	PF00856	chr8	26960795	26961013	219	73
MtSDG70	Medtr8g064320.1	PF00856	chr8	26961482	26962217	327	109
MtSDG71	Medtr8g070070.1	PF00856	chr8	29747371	29752183	2310	770
MtSDG72	Medtr8g070075.1	PF00856	chr8	29753285	29755561	2277	759
MtSDG73	Medtr8g071030.1	PF00856	chr8	30125511	30127760	2250	750
MtSDG74	Medtr8g078505.1	PF00856	chr8	33485570	33488636	1602	534
MtSDG75	Medtr8g078490.1	PF00856	chr8	33514038	33522768	3762	1254
MtSDG76	Medtr8g105200.1	PF00856	chr8	44366067	44369752	1449	483
MtSDG77	Medtr0071s0100.1	PF00856	scaffold0071	53704	63675	1158	386
MtSDG78	Medtr0376s0030.1	PF00856	scaffold0376	8375	10111	1737	579
MtPRMT1	Medtr2g054880.2	PF05185	chr2	23470110	23476622	1125	375
MtPRMT2	Medtr3g108640.1	PF05185	chr3	50162533	50174758	1944	648
MtPRMT3	Medtr4g119900.1	PF05185	chr4	49684309	49691222	1605	535
MtJMJ1	Medtr1g008060.3	PF02373	chr1	708137	717720	5106	1702
MtJMJ2	Medtr1g038400.1	PF02373	chr1	14138656	14141615	1716	572
MtJMJ3	Medtr1g038370.1	PF02373	chr1	14154078	14157394	1683	561
MtJMJ4	Medtr1g069565.2	PF02373	chr1	30198834	30209280	2844	948
MtJMJ5	Medtr1g071300.2	PF02373	chr1	31623300	31629331	1632	544
MtJMJ6	Medtr1g078070.1	PF02373	chr1	34920281	34930614	3696	1232
MtJMJ7	Medtr1g083120.1	PF02373	chr1	36993703	37001873	2844	948
MtJMJ8	Medtr1g090763.1	PF02373	chr1	40778826	40779221	396	132
MtJMJ9	Medtr1g094740.1	PF02373	chr1	42579741	42588916	4446	1482
MtJMJ10	Medtr1g114070.1	PF02373	chr1	51454157	51464605	2964	988
MtJMJ11	Medtr1g114130.1	PF02373	chr1	51478339	51487383	3249	1083
MtJMJ12	Medtr1g114150.1	PF02373	chr1	51495110	51508406	3180	1060
MtJMJ13	Medtr2g011630.3	PF02373	chr2	2845458	2851462	2910	970

MtJMJ14	Medtr2g024140.1	PF02373	chr2	8590982	8594447	2070	690
MtJMJ15	Medtr2g024240.1	PF02373	chr2	8672771	8676574	1185	395
MtJMJ16	Medtr2g024270.1	PF02373	chr2	8718834	8722997	2481	827
MtJMJ17	Medtr2g047130.2	PF02373	chr2	20672347	20678070	3012	1004
MtJMJ18	Medtr3g075210.1	PF02373	chr3	34214998	34224724	4719	1573
MtJMJ19	Medtr3g077420.1	PF02373	chr3	34751208	34755524	1488	496
MtJMJ20	Medtr3g099370.1	PF02373	chr3	45540890	45551717	2934	978
MtJMJ21	Medtr4g066020.1	PF02373	chr4	24877856	24885444	1242	414
MtJMJ22	Medtr4g091520.1	PF02373	chr4	36263391	36270275	3849	1283
MtJMJ23	Medtr4g132540.2	PF02373	chr4	55409367	55414991	2613	871
MtJMJ24	Medtr5g010300.1	PF02373	chr5	2711113	2733852	5499	1833
MtJMJ25	Medtr5g029370.1	PF02373	chr5	12300622	12307715	2538	846
MtJMJ26	Medtr5g047620.1	PF02373	chr5	20864740	20870294	2493	831
MtJMJ27	Medtr5g065200.1	PF02373	chr5	27394826	27402110	2514	838
MtJMJ28	Medtr7g407000.1	PF02373	chr7	1037796	1039047	465	155
MtJMJ29	Medtr7g097070.1	PF02373	chr7	38999118	39006308	2523	841
MtJMJ30	Medtr7g106990.1	PF02373	chr7	43587759	43589114	1329	443
MtJMJ31	Medtr7g117445.2	PF02373	chr7	48578247	48589222	2898	966
MtJMJ32	Medtr8g465720.1	PF02373	chr8	23439286	23440080	498	166
MtJMJ33	Medtr8g089260.1	PF02373	chr8	37084218	37088931	3138	1046
MtJMJ34	Medtr0020s0100.1	PF02373	scaffold0020	134188	138552	2166	722
MtHDMA1	Medtr1g023510.1	PF04433	chr1	7537822	7546911	5808	1936
MtHDMA2	Medtr1g050535.2	PF04433	chr1	19554387	19559654	2661	887
MtHDMA3	Medtr3g055090.1	PF04433	chr3	21645628	21648360	2247	749
MtHDMA4	Medtr3g088480.1	PF04433	chr3	40113660	40120664	2349	783
MtHDMA5	Medtr3g099000.1	PF04433	chr3	45370551	45376390	1623	541
MtHDMA6	Medtr3g113170.1	PF04433	chr3	52876598	52885239	5982	1994
MtHDMA7	Medtr3g116120.1	PF04433	chr3	54291302	54294108	1452	484
MtHDMA8	Medtr4g069830.1	PF04433	chr4	26258593	26267090	3126	1042
MtHDMA9	Medtr5g085170.1	PF04433	chr5	36752674	36753975	1302	434
MtHDMA10	Medtr6g053260.1	PF04433	chr6	19123038	19126671	2127	709
MtHDMA11	Medtr6g084700.1	PF04433	chr6	31822987	31825649	2439	813
MtHDMA12	Medtr8g464450.2	PF04433	chr8	22767746	22771406	2262	754
MtHAG1	Medtr1g018030.2	PF00583	chr1	5159648	5161518	579	193
MtHAG2	Medtr1g049370.1	PF00583	chr1	19057960	19059200	702	234
MtHAG3	Medtr1g095000.1	PF00583	chr1	42763908	42766828	843	281
MtHAG4	Medtr1g103110.1	PF00583	chr1	46647824	46648731	573	191
MtHAG5	Medtr1g103150.1	PF00583	chr1	46655675	46657091	573	191
MtHAG6	Medtr1g107255.1	PF00583	chr1	48542831	48547230	1692	564
MtHAG7	Medtr2g041620.1	PF00583	chr2	18274584	18277891	900	300
MtHAG8	Medtr3g030055.1	PF00583	chr3	9501265	9502343	468	156
MtHAG9	Medtr3g034200.1	PF00583	chr3	11036986	11042635	552	184
MtHAG10	Medtr3g050860.1	PF00583	chr3	20039441	20042747	1089	363
MtHAG11	Medtr3g463610.1	PF00583	chr3	25502412	25503737	660	220
MtHAG12	Medtr3g465010.1	PF00583	chr3	26263913	26265787	1167	389
MtHAG13	Medtr3g083200.1	PF00583	chr3	37548338	37552754	612	204
MtHAG14	Medtr3g093540.1	PF00583	chr3	42746950	42749380	771	257
MtHAG15	Medtr3g103110.2	PF00583	chr3	47520766	47524276	2403	801
MtHAG16	Medtr3g103120.1	PF00583	chr3	47527057	47529588	2532	844
MtHAG17	Medtr3g103160.1	PF00583	chr3	47549686	47551256	1335	445

MtHAG18	Medtr4g007130.1	PF00583	chr4	962439	965131	1230	410
MtHAG19	Medtr4g046047.1	PF00583	chr4	16076770	16078121	465	155
MtHAG20	Medtr4g095700.1	PF00583	chr4	39917595	39919629	1236	412
MtHAG21	Medtr4g099530.1	PF00583	chr4	41342856	41345368	1548	516
MtHAG22	Medtr4g122030.1	PF00583	chr4	50385405	50387046	1053	351
MtHAG23	Medtr4g129490.1	PF00583	chr4	53942577	53949233	798	266
MtHAG24	Medtr5g006450.1	PF00583	chr5	923125	924542	573	191
MtHAG25	Medtr5g006460.1	PF00583	chr5	925743	926338	582	194
MtHAG26	Medtr5g006470.1	PF00583	chr5	927842	928661	567	189
MtHAG27	Medtr5g006510.1	PF00583	chr5	935138	935780	576	192
MtHAG28	Medtr5g006520.1	PF00583	chr5	938994	948297	5727	1909
MtHAG29	Medtr5g007400.1	PF00583	chr5	1339606	1343203	774	258
MtHAG30	Medtr5g015300.1	PF00583	chr5	5283409	5288583	1830	610
MtHAG31	Medtr5g015810.1	PF00583	chr5	5544338	5546827	1212	404
MtHAG32	Medtr5g024390.1	PF00583	chr5	9819369	9820655	1287	429
MtHAG33	Medtr5g024500.1	PF00583	chr5	9853325	9854611	1287	429
MtHAG34	Medtr5g035040.1	PF00583	chr5	15245870	15248625	1131	377
MtHAG35	Medtr5g035060.1	PF00583	chr5	15250417	15253927	900	300
MtHAG36	Medtr5g080780.1	PF01853	chr5	34590396	34594554	1401	467
MtHAG37	Medtr5g098790.1	PF00583	chr5	43263342	43266567	495	165
MtHAG38	Medtr6g072100.1	PF00583	chr6	26706231	26710199	792	264
MtHAG39	Medtr7g024760.1	PF00583	chr7	8169263	8169963	531	177
MtHAG40	Medtr7g024765.1	PF00583	chr7	8171538	8172663	534	178
MtHAG41	Medtr7g024780.1	PF00583	chr7	8176955	8177485	531	177
MtHAG42	Medtr7g073990.1	PF00583	chr7	27613857	27617487	738	246
MtHAG43	Medtr7g084140.1	PF00583	chr7	32440227	32443905	597	199
MtHAG44	Medtr7g096040.1	PF00583	chr7	38499025	38508927	1629	543
MtHAG45	Medtr7g104610.1	PF00583	chr7	42399253	42399714	462	154
MtHAG46	Medtr7g109640.1	PF00583	chr7	44870216	44872606	2391	797
MtHAG47	Medtr7g114410.1	PF00583	chr7	47234493	47235146	534	178
MtHAG48	Medtr7g117040.1	PF00583	chr7	48323136	48326276	525	175
MtHAG49	Medtr8g062330.1	PF00583	chr8	26044640	26045983	1344	448
MtHAG50	Medtr0439s0020.1	PF00583	scaffold0439	1919	5522	669	223
MtHAG51	Medtr2102s0010.1	PF00583	scaffold2102	285	839	555	185
MtHAM1	Medtr3g007710.1	PF01853	chr3	1129490	1136078	1323	441
MtHAC1	Medtr1g047930.1	PF08214	chr1	18133654	18135721	1125	375
MtHAC2	Medtr3g101100.1	PF08214	chr3	46518191	46524451	4188	1396
MtHAC3	Medtr3g107960.2	PF08214	chr3	49877612	49884336	3621	1207
MtHAC4	Medtr4g005050.2	PF08214	chr4	25942	38639	5130	1710
MtHAC5	Medtr5g017020.1	PF08214	chr5	6160396	6163833	1683	561
MtHAC6	Medtr5g085310.1	PF08214	chr5	36846047	36850749	3078	1026
MtHAC7	Medtr6g017000.1	PF08214	chr6	6665635	6670322	2844	948
MtHAC8	Medtr6g017010.1	PF08214	chr6	6678099	6681722	2310	770
MtHAC9	Medtr6g017120.1	PF08214	chr6	6765240	6765911	462	154
MtHAC10	Medtr6g017135.1	PF08214	chr6	6774192	6775745	432	144
MtHAC11	Medtr6g017140.1	PF08214	chr6	6778377	6780888	1362	454
MtHAF1	Medtr5g026660.1	PF09247	chr5	10995785	11016376	5709	1903
MtHDA1	Medtr1g016440.1	PF00850	chr1	4371842	4377658	1053	351
MtHDA2	Medtr2g087270.1	PF00850	chr2	36677137	36680260	1161	387

MtHDA3	Medtr3g077120.1	PF00850	chr3	34630898	34631660	579	193
MtHDA4	Medtr3g077160.1	PF00850	chr3	34636818	34643051	1431	477
MtHDA5	Medtr3g118535.1	PF00850	chr3	55483175	55497394	1497	499
MtHDA6	Medtr4g055080.1	PF00850	chr4	20022768	20032485	1293	431
MtHDA7	Medtr4g109500.1	PF00850	chr4	45499722	45507261	1968	656
MtHDA8	Medtr4g111985.1	PF00850	chr4	46328880	46339481	1923	641
MtHDA9	Medtr5g004640.2	PF00850	chr5	45920	52671	1488	496
MtHDA10	Medtr8g093800.1	PF00850	chr8	39246652	39251957	1263	421
MtSRT1	Medtr3g460800.1	PF02146	chr3	23895713	23905992	1416	472
MtSRT2	Medtr3g096710.1	PF02146	chr3	44259211	44265766	1152	384
MtHDT1	Medtr2g084815.2	HDT	chr2	35979101	35982483	909	303
MtHDT2	Medtr4g055440.1	HDT	chr4	20198703	20202553	804	268
MtHDT3	Medtr4g055450.1	HDT	chr4	20204358	20207970	1083	361
